# Do cancer risk and benefit–harm ratios influence women’s consideration of risk-reducing mastectomy? A scenario-based experiment in five European countries

**DOI:** 10.1371/journal.pone.0218188

**Published:** 2019-06-12

**Authors:** Felix G. Rebitschek, Nora Pashayan, Martin Widschwendter, Odette Wegwarth

**Affiliations:** 1 Harding Center for Risk Literacy, Max Planck Institute for Human Development, Berlin, Germany; 2 Department of Applied Health Research, University College London, London, United Kingdom; 3 Department of Women’s Cancer, University College London, London, United Kingdom; 4 Center for Adaptive Rationality, Max Planck Institute for Human Development, Berlin, Germany; Ohio State University Wexner Medical Center, UNITED STATES

## Abstract

**Background:**

Personal cancer risk assessments enable stratified care, for example, offering preventive surgical measures such as risk-reducing mastectomy (RRM) to women at high risk for breast cancer. In scenario-based experiments, we investigated whether different benefit–harm ratios of RRM influence women’s consideration of this, whether this consideration is influenced by women’s perception of and desire to know their personal cancer risk, or by their intention to take a novel cancer risk-predictive test, and whether consideration varies across different countries.

**Method:**

In January 2017, 1,675 women 40 to 75 years of age from five European countries—Czech Republic, Germany, UK, Italy, and Sweden—took part in an online scenario-based experiment. Six different scenarios of hypothetical benefit–harm ratios of RRM were presented in accessible fact box formats: Baseline risk/risk reduction pairings were 20/16, 20/4, 10/8, 10/2, 5/4, and 5/1 out of 1,000 women dying from breast cancer.

**Results:**

Varying the baseline risk of dying from breast cancer and the extent of risk reduction influenced the decision to consider RRM for 23% of women. Decisions varied by country, risk perception, and the intention to take a cancer risk-predictive test. Women who expressed a stronger intention to take such a test were more likely to consider having RRM. The desire to know one’s risk of developing any female cancer in general moderated women’s decisions, whereas the specific desire to know the risk of breast cancer did not.

**Conclusions:**

In this hypothetical scenario-based study, only for a minority of women did the change in benefit–harm ratio inform their consideration of RRM. Because this consideration is influenced by risk perception and the intention to learn one’s cancer risks via a cancer risk-predictive test, careful disclosure of different potential preventive measures and their benefit–harm ratios is necessary before testing for individual risk. Furthermore, information on risk testing should acknowledge country-specific sensitivities for benefit–harm ratios.

## Introduction

Genetic, epigenetic, lifestyle, and reproductive factors alone or combined can be used to predict a woman’s risk for developing breast cancer.[1–3] Although the average 10-year absolute risk of breast cancer in women in the UK aged 50 years is 2.85%, women at the lowest and highest percentile of the risk distribution have a 0.53% and 9.96% 10-year risk, respectively.[4] Risk prediction can be used in each of these cases to enable risk-stratified early detection and risk-reducing preventive interventions.[5–7]

The FORECEE Consortium (Female Cancer Prediction Using Cervical Omics To Individualise Screening And Prevention; https://forecee.eu) aims to develop a risk-predictive test—for breast, cervical, endometrial, and ovarian cancer—in cervical epithelial cells, based on epigenetic markers.[8] Once identified, women at high risk of breast cancer could be offered risk-reducing surgery. Although there is no evidence from randomised clinical trials on the effectiveness of risk-tailored preventive intervention at the population level, there are clinical guidelines that suggest the option of risk-reducing surgery, for example, for women with *BRCA1* or *BRCA2* mutations.[9] Such interventions have both potential benefits and harms (e.g., psychological distress, complications from surgery).[10]

Among *BRCA* mutation carriers in Europe, more recent findings reveal proportions of about 36% in the Netherlands[11] and 34% in Wales[12] who opted for a risk-reducing mastectomy (RRM). Known factors that influence RRM decisions are having children, risk perception, cancer worry, and risk level.[13] What happens, however, if women misperceive the numerical relationship between their individual cancer risk and the potential benefit from risk reduction, as is the case for cancer screening?[14] Women who overestimate benefit–harm ratios may more likely consider mastectomy.[15] This may be even more pronounced for women who already have an elevated perception of their individual breast cancer risk.[16, 17] Moreover, women who have a strong desire to know their cancer risks and who perceive being in control of managing these risks[18] may also be more likely to control any elevated breast cancer mortality risk effectively by preventive surgery.[19]

Presenting women with six different scenarios, we investigated in this experimental survey study whether (1) varying baseline risks of breast cancer-specific mortality and varying extents of reduction in breast cancer-specific mortality following mastectomy would affect women’s consideration of RRM. In order to control whether aspects other than risk information would moderate their decisions, we further investigate if potential differences in women’s considerations are associated with (2) their perception of the individual female cancer risk, (3) their desire to know their female cancer risks, and (4) their intention to take a novel risk-predictive test if available. Finally, we investigated (5) whether the proportion of women considering RRM varied across European countries.

## Materials and methods

We developed an online survey questionnaire, which was translated into five languages. FORECEE Consortium[8] members from each of the five countries checked for the correctness and completeness of the translation. The survey was administered via e-mail by the market research institute Harris Interactive (Germany) to a sample of the Harris Interactive Panel and the Toluna Panel (sampling frame details in S1 Text).

The study was approved by the Institutional Ethics Board of the Max Planck Institute for Human Development, Berlin (Germany). It was carried out in accordance with the guidelines and regulations of the Max Planck Society for the Advancement of Science in Germany. Written informed consent was required to participate in this particular study.

In January 2017, Harris Interactive drew national samples of women in the core age group of screening programs and around that core age (aged 40 to 75 years) from five European countries that represent Northern (Sweden), Eastern (Czech Republic), Southern (Italy), Western (United Kingdom), and Central Europe (Germany): Of the 3,629 women invited (inclusion criteria: aged 40 to 75 years), 2,092 responded and 1,675 completed the survey (response rate: 61.4%). The questionnaire did not allow for item nonresponse. Sample selection and sample population are detailed elsewhere.[20]

### Survey questionnaire

**Fig 1:**
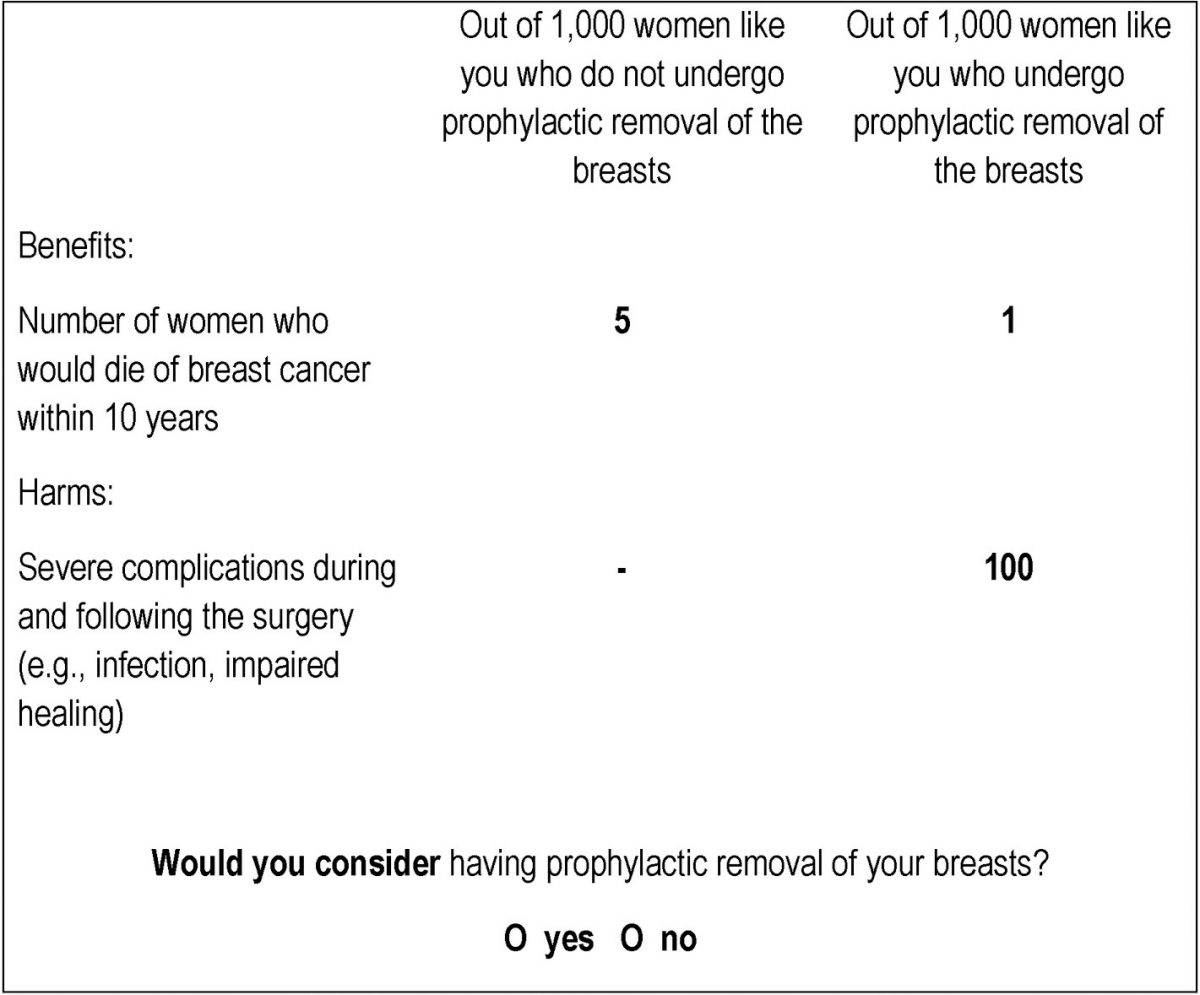
Example of a fact box which provides women with information on the benefits and harms of having/not having RRM. Note that although the information on the benefits was varied across scenarios, the information on harms remained constant.

The survey consisted of three sections eliciting women’s disease risk perception, their attitude toward cancer risk-predictive testing based on epigenetic markers, and their considerations to undertake RRM. Participants were presented with a leaflet containing baseline risk information about developing breast, cervical, endometrial, or ovarian cancer within 10 years and a novel risk-predictive test (S1 Fig) before they were asked once more about their disease risk perceptions. Subsequently, participants were presented with an introduction to six hypothetical scenarios that asked them to imagine having a higher-than-average risk of breast cancer (S2 Fig). They were then presented with a fact box informing them about the fictitious effect of having or not having RRM on breast cancer mortality risk (Fig 1). Fact boxes are tabular formats that present information on the benefits and harms of a medical option for a control group and an intervention group in absolute numbers, which are adjusted to the same denominator.[21–23]

Within the fact box, in a frequency format,[24] information was provided on the potential benefits and harms of RRM. Numbers characterising the benefit were varied experimentally: The baseline risks of 10-year breast cancer-related mortality (defined interval instead of lifetime risk[25]) were varied from high to extremely high across the scenarios (20 vs. 10 vs. 5 out of 1,000 women not having RRM). Following RRM, each breast cancer mortality risk was reduced by 20% and 80% to represent limited risk reduction and realistic risk reduction, respectively. None of these variations led any scenario to be more realistic than another from the perspective of the participants. In fact, prophylactic mastectomy could reduce the risk of developing breast cancer by about 90% to 95%[26] and breast cancer mortality by 80%.[27]

The fact box was presented together with the question “Would you consider having prophylactic removal of your breasts?”. At the end of the survey, participants were debriefed that the figures in the scenarios were hypothetical and how they were varied across the scenarios.

### Analysis

Within-subjects and between-subjects analyses were performed using variance analyses, with samples approximating normal distributions. Linear regressions with the dependent variable average level of mastectomy consideration across the six scenarios and logistic regressions with the dependent variable scenario-specific mastectomy consideration were conducted to investigate the influences of disease risk perception, a general desire to know one’s female cancer risk, and the intention to have a risk-predictive test for female cancer. Different response patterns of consideration were identified and the association with the study variables was assessed using the Chi-squared test.

## Results

Among the 1,675 women in the study (the participants characteristics are described in S1 Table), we identified three general response patterns (Table 1): 968 (58%) did not consider mastectomy in any of the presented scenarios, 323 (19%) decided to consider mastectomy in each of the six scenarios, and 384 (23%) women varied their responses across the scenarios. Thus, only for the latter group does the numerical relationship between cancer risks and risk reduction through RRM appear to have an effect on their decisions to consider mastectomy. Notably, 112 out of 384 participants violated transitivity across scenarios at least once: 46 considered a mastectomy for reducing mortality risk from 20 to 12, but not for a reduction from 20 to 4 out of 1,000; 53 participants considered it for 10 to 6, but not for 10 to 2; and 41 participants considered it for 5 to 3, but not for 5 to 1.

**Table 1:** Response patterns to the scenarios stratified by factors.

	Proportion: always no (*n* = 968) [%]	Proportion: scenario- dependent (*n* = 384) [%]	Proportion: always yes (*n* = 323) [%]	*p*^a^
Age [groups]				.909
40–49 years	56.4	23.0	20.6	
50–59 years	58.8	23.3	18.0	
60–69 years	58.9	21.7	19.4	
70–75 years	56.1	25.0	18.9	
Education [ICED]				< .001
High	50.6	30.0	19.3	
Medium	62.7	19.8	17.5	
Low	55.3	22.4	22.4	
Personal female cancer history				.041
Yes	48.1	23.1	28.8	
No	58.6	23.0	18.4	
Not known	48.0	20.0	32.0	
Reporting personal or familial cancer history				.672
Reporting	57.9	23.4	18.7	
Not reporting	57.7	22.1	20.3	
Country				< .001
Czech Republic	61.8	22.8	15.4	
Germany	75.5	15.2	9.3	
UK	42.1	33.1	24.8	
Italy	65.1	14.8	20.1	
Sweden	43.0	29.4	27.6	
Breast cancer risk perception				.008
Increased	49.9	24.8	25.4	
Correct	59.6	22.8	17.7	
Decreased	60.4	21.8	17.8	
Desire to know one’s female cancer risks				< .001
All risks	51.5	25.7	22.8	
Some risks	58.7	28.0	13.3	
No risks	72.1	14.8	13.1	
Desire to know one’s breast cancer risk				
Yes	52.5	25.8	21.7	< .001
No	71.3	15.6	13.1	
Intention to take a novel risk-predictive test				< .001
Definitely DO	48.3	26.0	25.8	
Probably DO	56.4	25.8	17.8	
Probably NOT	69.7	15.3	15.0	
Definitely NOT	76.8	10.7	12.5	

^a^Chi-squared tests

The distribution of response patterns varied not with age, but with education (*χ*^**2**^(4) = 23.11, *p* < .001): Women with low levels of the International Standard Classification of Education (ISCED) were more likely to consider mastectomy in every scenario, those with medium levels were more likely to never consider mastectomy, and those with high levels were more likely to consider mastectomy in dependence of the risk information provided in the scenarios. Response patterns varied by country (*χ*^**2**^(8) = 124.07, *p* < .001): 75.5% of women in Germany but only 42.1% of women in the United Kingdom and 43.0% in Sweden never considered mastectomy in the scenarios. Conversely, 24.8% of women in the United Kingdom and 27.6% in Sweden, but only 9.3% of women in Germany, considered mastectomy in each scenario (Table 1). Women reporting personal or familial cancer experience did not respond differently from women without this experience, but a higher proportion of women with personal female cancer experience considered mastectomy across all scenarios.

### Is the numerical relationship between breast cancer mortality risks and risk reduction across the scenarios associated with the consideration of RRM?

**Fig 2:**
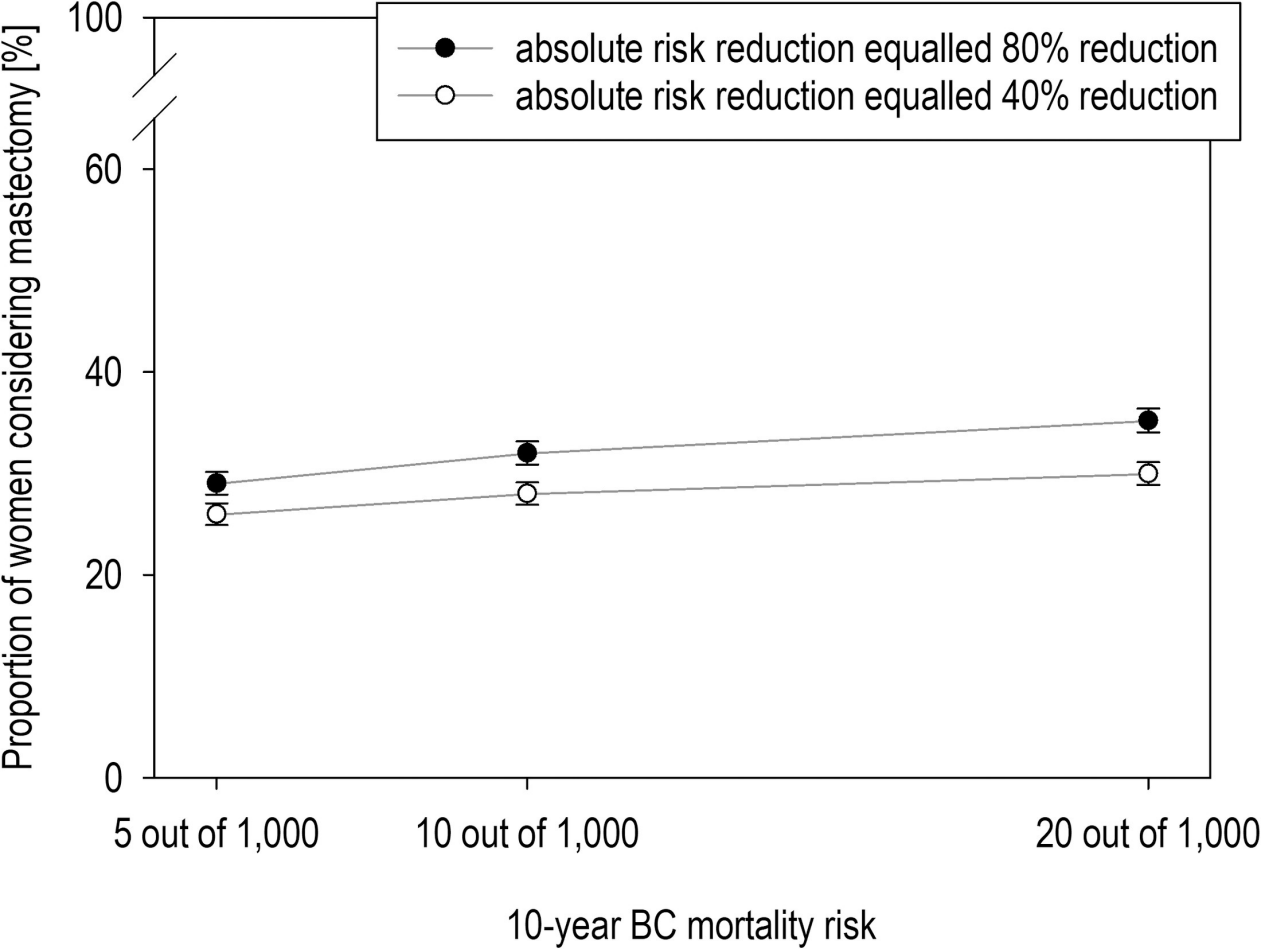
Mastectomy considerations according to baseline mortality risks. The risk of dying from breast cancer and the level of risk reduction were varied across the scenarios. Error bars show the standard error of the mean.

Higher baseline risk of breast cancer mortality (*F*(2,3348) = 43.67, *p* < .001, *η*_p_^2^ = 0.03) and larger mortality risk reduction with RRM (*F*(1,1674) = 62.86, *p* < .001, *η*_p_^2^ = 0.04) were associated with a larger average consideration of RRM in our sample (Fig 2). The effects remained stable after excluding from the analysis women with female cancer history (*n* = 104), breast cancer diagnosis, or ductal carcinoma in situ (*n* = 59). Including age group as a between-subject factor revealed neither a main effect on consideration (*F*(3,1671) = 0.25, *p* = .862) nor interactions with baseline risk (*F*(6,3243) = 1.81, *p* = .095) or risk reduction (*F*((3,1671) = 2.19, *p* = .087). Factors associated with scenario-dependent responses are presented in S2 Table.

### Does the consideration of RRM vary across European countries?

The decisions on considering RRM were influenced by multiple factors, including country, level of education, personal female cancer history, breast cancer risk perception after reading an information leaflet, desire to know one’s female cancer risks, desire to know one’s breast cancer risk, and intention to take a novel risk-predictive test (Table 2).

**Table 2:** Average mastectomy consideration stratified by factors.

	*n*	Average mastectomy consideration M(SD)	*F*	*p*	*η*_p_^2^ ^a^
Age [groups]			*F*(3, 1671) = 0.25	.862	–
40–49 years	495	0.31 (0.41)			
50–59 years	490	0.29 (0.40)			
60–69 years	494	0.29 (0.41)			
70–75 years	196	0.30 (0.40)			
Education [ICED]			*F*(2, 1672) = 5.37	.005	0.01
High	393	0.33 (0.41)			
Medium	817	0.27 (0.40)			
Low	465	0.30 (0.41)			
Personal female cancer history			*F*(2, 1672) = 4.49	.011	0.01
Yes	104	0.40 (0.45)			
No	1,546	0.29 (0.40)			
Not known	25	0.43 (0.47)			
Country			*F*(4, 1670) = 27.58	< .001	0.06
Czech Republic	356	0.25 (0.38)			
Germany	335	0.16 (0.32)			
UK	323	0.42 (0.43)			
Italy	338	0.27 (0.41)			
Sweden	323	0.42 (0.43)			
Breast cancer risk perception			*F*(2, 1672) = 8.78	< .001	0.01
Increased	343	0.38 (0.44)			
Correct	905	0.28 (0.40)			
Decreased	427	0.28 (0.40)			
Desire to know one’s female cancer risks			*F*(2, 1672) = 28.03	< .001	0.03
All risks	1,066	0.36 (0.42)			
Some risks	150	0.24 (0.37)			
No risks	459	0.19 (0.36)			
Desire to know one’s breast cancer risk			*F*(1, 1673) = 45.18	< .001	0.03
Yes	1,201	0.34 (0.36)			
No	474	0.19 (0.36)			
Intention to take a novel risk-predictive test			*F*(3, 1671) = 18.17	< .001	0.03
Definitely DO	497	0.39 (0.44)			
Probably DO	759	0.30 (0.40)			
Probably NOT	307	0.21 (0.37)			
Definitely NOT	112	0.16 (0.34)			

^a^ *η*_p_^2^ (Partial eta square) reflects the ratio of variance associated with an effect to the sum of this variance plus associated error variance.

**Fig 3:**
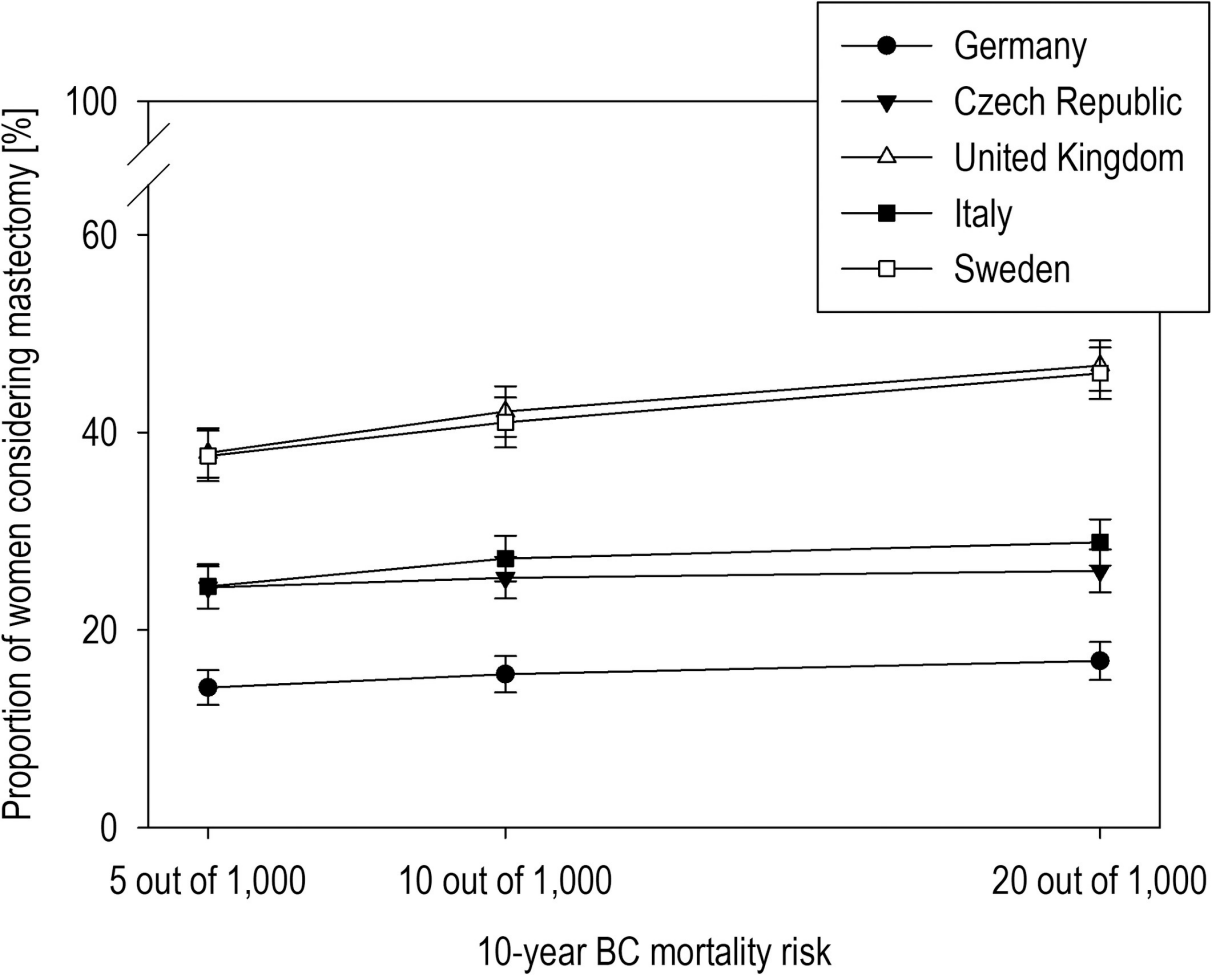
European mastectomy considerations according to baseline mortality risks. Proportion of women considering RRM by country and baseline risk of breast cancer-specific mortality. Error bars show the standard error of the mean.

As the response patterns indicated, women’s decisions varied across countries. For each scenario, women from the United Kingdom and Sweden were most likely to consider having RRM, whereas women from Germany were least likely to consider having it (Fig 3).

**Fig 4:**
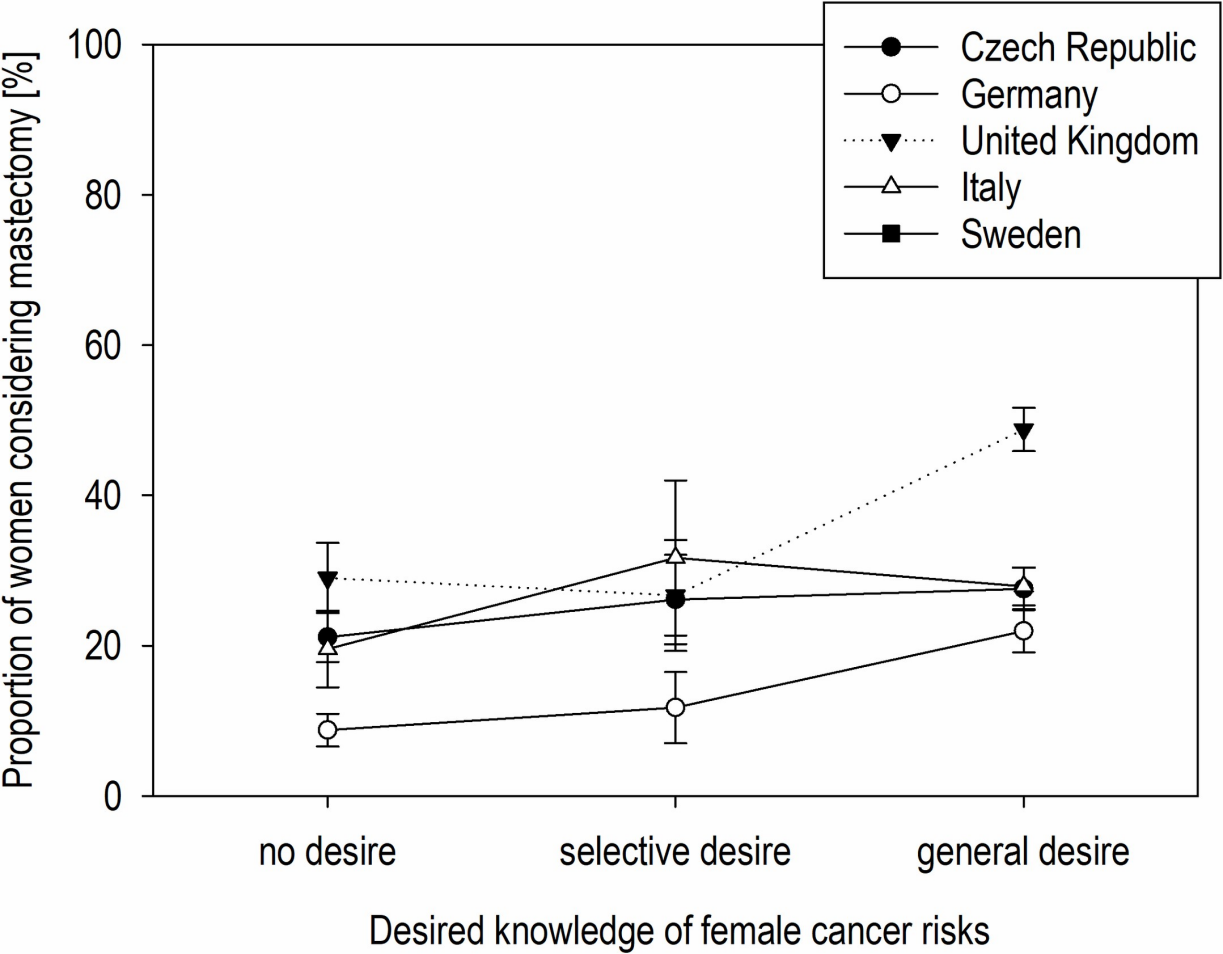
Mastectomy considerations according to country and general desire to know one's cancer risks. Proportion of women considering RRM by country and the amount of cancer risk information desired. Error bars show the standard error of the mean.

A stepwise regression showed that country-specific considerations of mastectomy were moderated by the general desire to know one’s female cancer risks (*F*(8, 1666) = 34.22, *p* < .001, *R*^2^ = 0.10) (Fig 4). The general desire to know (*β* = .22, *p* < .001) explained the increased consideration of mastectomy across countries. However, women from Italy (*β* = -0.18, *p* < .001), Germany (*β* = -0.09, *p* = .026), and the Czech Republic (*β* = -0.16, *p* < .001) with a general desire to know their female cancer risks were less likely to consider mastectomy than those from the United Kingdom. Moreover, German women (*β* = -.13, *p* < .001) were less likely to consider mastectomy in general (Fig 4).

### Does an increased risk perception of disease influence consideration?

During the study, the women were given a leaflet containing the age-specific risks of developing breast cancer.[20] After reading it, they were asked to indicate an estimate of their personal breast cancer risk. The proportion of women who estimated having an increased risk of breast cancer was 20.5%. Women who perceived themselves to be at higher risk considered RRM more often (averaged across the scenarios *M* = 0.38, *SD* = 0.44). In comparison, the average consideration of those who did not perceive themselves to be at higher risk was *M* = 0.28 (*SD* = 0.40). This association holds after controlling for several factors, including the desire to know one’s risk level and the intention to take a novel risk-predictive test under development.

### Is women’s intention to take a novel cancer risk-predictive test associated with their consideration of RRM?

The stepwise regression showed that the women were more likely to decide to consider mastectomy when they intended to take the predictive test for four different types of cancer and when they had a general desire to know their cancer risk, averaged across the four types of cancer. At the same time, when controlled for the general desire to know, the desire to know one’s breast cancer risk did not make it more likely to consider mastectomy (Table 3). As women’s disease risk perception may have interfered with their perceptions of the given mortality risks across the scenarios, it is noteworthy that the described effect of the general desire to know held even when controlled for disease risk perception.

**Table 3:** Country-specific linear regressions of average consideration of RRM.

	Standardised coefficient [95% Confidence interval] (*p*)					
	Czech Republic	Germany	United Kingdom	Italy	Sweden	All countries
Risk perception of BC (increased estimation after reading information leaflet)^a^	0.07 [-0.02,0.17] (.184)	-0.05 [-0.15,0.05] (.386)	0.22 [0.11,0.34] (.001)	0.09 [-0.01,0.20] (.092)	0.07 [-0.05,0.18] (.242)	0.10 [0.05,0.15] (< .001)
Desire to know one’s breastcancer risk ^a^	0.09 [0.00,0.17] (.114)	0.17 [0.10,0.24] (.002)	0.16 [0.05,0.27] (.005)	0.04 [-0.07,0.16] (.430)	0.22 [0.11,0.33] (< .001)	0.16 [0.11,0.20] (< .001)
^b^	0.08 [-0.18,0.35] (.641)	-0.05 [-0.27,0.17] (.767)	-0.26 [-0.58,0.07] (.118)	-0.07 [-0.43,0.30] (.696)	-0.09 [-0.42,0.23] (.568)	-0.09 [-0.22, 0.05] (.248)
General desire to know one’s cancer risks ^a^	0.08 [-0.01,0.17] (.131)	0.19 [0.12,0.26] (.001)	0.20 [0.09,0.31] (< .001)	0.05 [-0.07,0.17] (.333)	0.24 [0.13,0.35] (< .001)	0.18 [0.13,0.23] (< .001)
^c^	0.08 [-0.01,0.17] (.142)	0.19 [0.07,0.31] (.001)	0.20 [0.09,0.31] (< .001)	0.06 [-0.06,0.19] (.304)	0.24 [0.12,0.35] (< .001)	0.17 [0.13,0.22] (< .001)
Intention to have a novel risk-predictive test ^a^	0.03 [-0.02,0.07] (.567)	0.20 [0.17,0.24] (< .001)	0.21 [0.16,0.27] (< .001)	0.12 [0.07,0.18] (.025)	0.19 [0.14,0.25] (< .001)	0.17 [0.15,0.20] (< .001)
^c^	0.03 [-0.02,0.07] (.621)	0.21 [0.17,0.25] (< .001)	0.21 [0.16,0.26] (< .001)	0.13 [0.07,0.20] (.019)	0.19 [0.13,0.25] (.001)	0.17 [0.15,0.19] (< .001)

^a^Adjusted for age, education, female cancer experience

^b^If additionally adjusted for general desire to know one’s cancer risk

^c^If additionally adjusted for BC risk perception.

To summarise and structure all described relationships, S3 Table classifies participants according to a pipeline of their perception of breast cancer risk, their desire to know their female cancer risks, and their intention to take a novel risk-predictive test which is under development.

## Discussion

Only 23% of the participants varied their decisions on RRM according to a given baseline risk of breast cancer mortality and the expected risk reduction due to the mastectomy. In contrast, most of the women always applied the same response strategy, that is, always responded “yes” or “no” regardless of information provided. Although this phenomenon may hint to the fact that the presented scenarios did not translate well to the women in our study, it is worth noting that the consistent response patterns systematically varied across the five European countries. For instance, we saw the largest proportion of women who would not consider undergoing RRM in Germany and the largest proportion who would always consider it in the United Kingdom and Sweden. Thus, the proportion of consistent response patterns observed in our survey might be less an artefact of women not understanding the information provided than a general national attitude toward having preventive surgeries to reduce cancer risk. The observed variations in our study are indeed quite in line with real uptake rates in different countries.[28] In Wales (UK), 34% of all *BRCA* mutation carriers identified up to 2015 underwent RRM[12], and 40% of participants opted for bilateral RRM in a study in England.[29] US data similarly indicate increasing acceptance of this preventive measure.[30] A study from the Netherlands shows that 36% of *BRCA* mutation carriers chose to undergo RRM within five years after disclosure of the test result.[11]

A history of large-scale prevention campaigning in the United Kingdom and Sweden[31] (with 42% of participants in each country considering RRM) could have promoted acceptance of preventive measures in a larger proportion of the population than within the health systems of the Czech Republic and Italy (with around 26% of participants in each country considering RRM). The low proportion of women (16%) considering RRM in Germany, on the other hand, could be due to media reporting on potential harms or shortcomings of personalized medicine, whereas in the UK media, the potential merits are emphasized.[32] Moreover, high-quality medical service is easily accessible in Germany even for people with low incomes (9 out of 10 people in Germany have a statutory health insurance). One can speculate that particularly women in Germany expect easy access to alternative preventive measures. Future research thus needs to compare breast cancer prevention across different countries with different healthcare systems.

Importantly, breast cancer risk perception, after reading an information leaflet on age-specific breast cancer disease risk, influenced the consideration of RRM, although the scenarios presented numbers on the risk of breast cancer mortality. Those who perceived themselves to be at higher risk of developing the disease were more likely to consider having mastectomy, even if after having available information about “their” breast cancer mortality risk. Providing this subgroup with particular care accompanied by evidence-based and consistent information at all stages thus appears crucial for ensuring informed decision-making.

The desire to know one’s breast cancer risk was not associated specifically with considering mastectomy in our study, whereas a general desire to know one’s risk of developing the four different female cancers and the intention to take an offered cancer risk-predictive test (even though it is still under development) were associated with considering. Many preventive considerations regarding breast cancer are not necessarily based on disease-related interest. Instead, it cannot be excluded that offering the test could nudge some part of the general population toward surgery by advancing cancer risk-testing opportunities in the first place.

The hypothetical scenario-based approach does not allow for a direct transfer of our findings to real-world health considerations. For instance, there is a gap between considering and having prophylactic mastectomy.[33] Furthermore, benefits and harms of RRM were selective and its effectiveness was varied. For example, to keep our experimental stimuli comprehensible, we did not include numbers for potential distress by having RRM. Instead, we expected that participants themselves would most likely anticipate potential distress of breast removal. It remains a limitation that we did not inquire about their feelings and perceived distress as this presumably factors in considering RRM.

Participants could only marginally experience the decision faced by a high-risk woman. However, we chose to use very high risks so that participants would not underestimate the impact of potential real-world risks. A limitation of the study is that only breast cancer mortality risk is considered and not the risk of developing breast cancer. Another limitation is that the presentation of the scenarios was in random sequence and non-adaptive, which can produce errors if participants are forced to make repeated judgments. This may have led the 112 participants (6.7%) to produce intransitive considerations. This is not surprising because low-numerate people have difficulties in understanding numerical risk information, but this could be overcome with the help of icon arrays in future studies.[34]

With a 61% response rate, there is a potential participation bias and nonresponse bias. However, there is unlikely to be bias by disease experience. For example, 3.5% of participants from Germany with a median age of 56 years reported a breast cancer diagnosis in the past. The cumulative risks of developing breast cancer in Germany for women aged 54 and 59 years are 2.9% and 4.1%, respectively.[35] Furthermore, our findings did not vary by including and excluding women who reported a personal history of female or breast cancer in our study. Generally, the potential for a selection bias by paid panellists as a sample is a qualitative limitation inherent to this type of online survey. To surmount such a limitation, future research should use computer-assisted telephone surveys with random digit dialling and representative cell-phone number shares.

How information is provided to the public is crucial. Obviously, many women in our study did not take the presented cancer mortality baseline risk into account and the extent of mortality reduction, but were influenced by other factors. It follows that, if risk-predictive tests for female cancers enter the healthcare system providing women with consistent evidence-based health information at all different stages of their care, this will be particularly important in order to ensure informed decisions. Furthermore, communication regarding cancer risk-predictive tests needs to promote reflection on potential benefits and harms of testing and non-testing as well as on risk management in the first place. Finally, communication strategies should also acknowledge country-specific health values.

## Supporting information

S1 Table Joint distributions for age and education in the populations and samples. (PDF)

S2 Table Predictors of scenario-based mastectomy consideration. (PDF)

S3 Table Hierarchically stratified consideration of mastectomy. (PDF)

S1 Fig The leaflet (English) with which participants were presented before the scenario study. (PDF)

S2 Fig The instruction of the task. (PDF)

S3 Fig Questionnaire used in the study (Czech version). (PDF)

S4 Fig Questionnaire used in the study (German version). (PDF)

S5 Fig Questionnaire used in the study (English version). (PDF)

S6 Fig Questionnaire used in the study (Italian version). (PDF)

S7 Fig Questionnaire used in the study (Swedish version). (PDF)

S1 Text Sampling frame and survey administration. (PDF)
