# Supplementary material for: Do cancer risk and benefit–harm ratios influence women’s consideration of risk-reducing mastectomy? A scenario-based experiment in five European countries
Source: PLoS One. 2019 Jun 12;14(6):e0218188. doi: 10.1371/journal.pone.0218188 (PMC6561593; doi:10.1371/journal.pone.0218188)
Supplement: S1 Table — (PDF) [file pone.0218188.s001.pdf]

S1 Table. Joint distributions for age and education in the populations and samples

	Education (ISCED)							
	Low		Medium		High		Total	
	Population [%]	Sample [%]	Population [%]	Sample [%]	Population [%]	Sample [%]	Population [%]	Sample [%]
	Czech Republic							
Age		n = 48		n = 256		n = 52		n = 356
40-49	10	8	32	30	40	38	30	28
50-59	22	25	27	25	30	27	27	25
60-69	41	63	29	35	20	23	30	37
70-75	27	4	12	11	10	12	14	10
	Germany							
Age		n = 63		n = 207		n = 65		n = 335
40-49	22	21	29	29	34	35	29	29
50-59	27	27	33	34	34	34	32	33
60-69	28	29	24	24	23	22	25	24
70-75	23	24	14	13	9	9	14	14
	United Kingdom							
Age		n = 96		n = 111		n = 116		n = 323
40-49	23	22	33	32	38	38	32	31
50-59	28	29	32	33	31	31	31	31
60-69	34	34	24	23	21	21	26	25
70-75	15	15	11	12	10	10	12	12
	Italy							
Age		n = 183		n = 112		n = 43		n = 338
40-49	23	21	42	40	48	42	33	30
50-59	26	25	35	34	28	28	30	28
60-69	31	38	18	20	19	23	25	30
70-75	19	16	5	6	5	7	13	12
	Sweden							
Age		n = 75		n = 131		n = 117		n = 323
40-49	16	13	32	32	39	38	31	30
50-59	23	23	31	31	29	31	29	29
60-69	39	47	26	27	24	24	28	30
70-75	22	17	10	10	8	8	12	11
