# Supplementary material for: Do cancer risk and benefit–harm ratios influence women’s consideration of risk-reducing mastectomy? A scenario-based experiment in five European countries
Source: PLoS One. 2019 Jun 12;14(6):e0218188. doi: 10.1371/journal.pone.0218188 (PMC6561593; doi:10.1371/journal.pone.0218188)
Supplement: S2 Table — (PDF) [file pone.0218188.s002.pdf]

S2 Table. **Predictors of scenario-based mastectomy consideration.**

Country	Factor	Scenario 5-3		Scenario 5-1		Scenario 10-6		Scenario 10-2		Scenario 20-12		Scenario 20-4	
		OR	*p*	OR	*p*	OR	*p*	OR	*P*	OR	*p*	OR	*P*
Czech Republic	Intention to have a risk-predictive test	-	-	-	-	-	-	-	-	-	-	-	-
	Female cancer experience	-	-	3.18	.013	-	-	-	-	-	-	-	-
	BC risk perception (increased estimation after information)	-	-	-	-	-	-	1.79	.037	-	-	-	-
	General desire to know one's cancer risks	-	-	-	-	-	-	-	-	-	-	-	-
	Education	1.68	.032	-	-	-	-	-	-	-	-	-	-
	Age group	-	-	-	-	-	-	-	-	-	-	-	-
	Desire to know one's BC risks	-	-	-	-	-	-	-	-	-	-	-	-
	BC risk perception x General desire to know one's cancer risks	-	-	-	-	-	-	-	-	-	-	-	-
	BC risk perception x Intention to have a risk-predictive test	-	-	-	-	-	-	-	-	-	-	-	-
Germany	Intention to have a risk-predictive test	1.76	.006	2.04	<.001	2.08	<.001	-	-	3.71	.001	-	-
	Female cancer experience	-	-	-	-	-	-	-	-	-	-	-	-
	BC risk perception (increased estimation after information)	-	-	-	-	-	-	-	-	-	-	-	-
	General desire to know one's cancer risks	-	-	-	-	-	-	3.07	.001	-	-	3.02	.001
	Education	1.95	.019	-	-	-	-	-	-	-	-	-	-
	Age group	-	-	-	-	-	-	-	-	-	-	-	-
	Desire to know one's BC risks	-	-	-	-	-	-	-	-	-	-	-	-
	BC risk perception x General desire to know one's cancer risks	-	-	-	-	-	-	-	-	-	-	-	-
	BC risk perception x Intention to have a risk-predictive test	-	-	-	-	-	-	-	-	-	-	-	-
United Kingdom	Intention to have a risk-predictive test	1.64	.002	-	-	1.62	.002	1.80	<.001	-	-	-	-
	Female cancer experience	-	-	-	-	-	-	-	-	-	-	-	-
	BC risk perception (increased estimation after information)	-	-	3.52	<.001	-	-	2.11	.012	-	-	2.05	.017
	General desire to know one's cancer risks	-	-	2.33	.004	-	-	-	-	2.52	.002	2.75	<.001

	Education	-	-	-	-	-	-	-	-	-	-	-	-
	Age group	-	-	-	-	-	-	-	-	-	-	-	-
	Desire to know one's BC risks	-	-	-	-	-	-	-	-	-	-	-	-
	BC disease-risk perception x General desire to know one's cancer risks	-	-	-	-	-	-	-	-	-	-	-	-
	BC disease-risk perception x Intention to have a risk-predictive test	1.39	<.001	-	-	1.39	.001	-	-	1.40	<.001	-	-
Italy	Intention to have a risk-predictive test	-	-	1.56	.016	-	-	1.56	.013	-	-	1.55	.011
	Female cancer experience	2.98	.020	-	-	-	-	-	-	-	-	-	-
	BC disease-risk perception (increased estimation after information)	-	-	-	-	-	-	1.91	.025	-	-	-	-
	General desire to know one's cancer risks	-	-	-	-	-	-	-	-	-	-	-	-
	Education	-	-	-	-	-	-	-	-	-	-	-	-
	Age group	-	-	1.33	.027	-	-	1.30	.038	-	-	1.30	.032
	Desire to know one's BC risks	-	-	-	-	-	-	-	-	-	-	-	-
	BC disease-risk perception x General desire to know one's cancer risks	-	-	-	-	-	-	-	-	-	-	-	-
	BC disease-risk perception x Intention to have a risk-predictive test	-	-	-	-	-	-	-	-	-	-	-	-
Sweden	Intention to have a risk-predictive test	-	-	-	-	-	-	-	-	-	-	-	-
	Female cancer experience			-	-	-	-	-	-	-	-	-	-
	BC disease-risk perception (increased estimation after information)	-	-	-	-	-	-	-	-	-	-	-	-
	General desire to know one's cancer risks	2.86	.001	2.37	.004	3.23	<.001	2.90	<.001	-	-	3.70	<.001
	Education	-	-	-	-	1.41	.030	-	-	-	-	-	-
	Age group	-	-	-	-	-	-	-	-	-	-	-	-
	Desire to know one's BC risks	-	-	-	-	-	-	-	-	-	-	-	-
	BC disease-risk perception x General desire to know one's cancer risks	-	-	-	-	-	-	-	-	-	-	-	-
	BC disease-risk perception x Intention to have a risk-predictive test	-	-	-	-	-	-	-	-	-	-	-	-

* The scenario label translates the baseline risk and the risk reduction in the respective scenario, e.g. scenario 5-3 means that 5 out of 1,000 women were shown to die from breast cancer, reducing this to 3 out of 1,000 by prophylactic mastectomy, scenario 20-4 means that 20 out of 1,000 women were shown to die from breast cancer, reducing this by 4 out of 1,000 by prophylactic mastectomy
