# Supplementary material for: Do cancer risk and benefit–harm ratios influence women’s consideration of risk-reducing mastectomy? A scenario-based experiment in five European countries
Source: PLoS One. 2019 Jun 12;14(6):e0218188. doi: 10.1371/journal.pone.0218188 (PMC6561593; doi:10.1371/journal.pone.0218188)
Supplement: S3 Table — (PDF) [file pone.0218188.s003.pdf]

S3 Table. Hierarchically stratified consideration of mastectomy.

Breast cancer risk perception	Proportion [%]	Desire to know one's female cancer risks[a]	Proportion [%]	Intention to take a novel risk-predictive test	Proportion [%]	Average mastectomy consideration[b] N	M(SD)
Increased	20.5	All female cancer risks	67.1	probably/ definitely	93.5	215	0.41(0.44)
				probably/ definitely NOT	*6.5* [c]	*15*	*0.53(0.46)*
		Breast cancer risk only	*2.9*	probably/ definitely	*90.9*	*10*	*0.22(0.34)*
				probably/ definitely NOT	*9.1*	*1*	*1.00(0.00)*
		No female cancer risk	24.8	probably/ definitely	36.5	31	0.39(0.42)
				probably/ definitely NOT	63.5	54	0.28(0.42)
Correct	54.0	All female cancer risks	64.5	probably/ definitely	95.5	558	0.34(0.42)
				probably/ definitely NOT	4.5	26	0.21(0.36)
		Breast cancer risk only	5.4	probably/ definitely	71.4	35	0.24(0.36)
				probably/ definitely NOT	*28.6*	*14*	*0.20(0.33)*
		No female cancer risk	25.9	probably/ definitely	26.9	63	0.22(0.37)
				probably/ definitely NOT	73.1	171	0.14(0.33)
Decreased	25.5	All female cancer risks	59.0	probably/ definitely	93.7	236	0.34(0.42)
				probably/ definitely NOT	6.3	*16*	*0.52(0.48)*
		Breast cancer risk only	*3.5*	probably/ definitely	*66.7*	*10*	*0.20(0.38)*
				probably/ definitely NOT	*33.3*	*5*	*0.10(0.22)*
		No female cancer risk	32.8	probably/ definitely	28.6	40	0.25(0.39)
				probably/ definitely NOT	71.4	100	0.12(0.30)

[a]Because desires to know only cervical, endometrial, and ovarian cancers are omitted, numbers do not add up to 100%. [b]Average consideration is calculated across the six scenarios presented. [c]Numbers in italics are based on small samples n<20.
