# Supplementary material for: Do cancer risk and benefit–harm ratios influence women’s consideration of risk-reducing mastectomy? A scenario-based experiment in five European countries
Source: PLoS One. 2019 Jun 12;14(6):e0218188. doi: 10.1371/journal.pone.0218188 (PMC6561593; doi:10.1371/journal.pone.0218188)
Supplement: S1 Fig — (PDF) [file pone.0218188.s004.pdf]

4C forecee
Four cancers
One test

The Women's Cancer Risk IDentification (WID) Test
A test under development to identify a woman's risk of developing breast cancer, ovarian cancer, cervical cancer, and womb cancer

Leaflet for women aged 50 years and above

the eve appeal
fighting women's cancers

Supported by the EC's Horizon 2020 Programme and The Eve Appeal
Copyright © 2015 FORECEE Consortium – All rights reserved

Women's cancer risk identification (WID) test

A test under development to identify a woman's risk of developing breast cancer, ovarian cancer, cervical cancer, and womb cancer

Why this leaflet?

With this leaflet we would like to inform you about a new test – the **w**omen's cancer risk **id**entification (WID) test – that is currently under development. This new test is meant to predict a **healthy** woman's risks of developing any of the following female cancers:
breast cancer, ovarian cancer, cervical cancer, and womb cancer.

You can also learn more about your baseline risk of getting any of these female cancers, the current procedures to reduce the threat of these cancers, and the potential benefits and harms of taking the WID test, which is currently under development.

What is your baseline risk of developing female cancers?

In order to better evaluate the potential value of this new test, it is helpful for you to understand an average woman's risk of developing any of these cancers. For the large majority of women, the risk of developing female cancers is fairly low. The following overview gives you an idea of how likely women at different ages will be diagnosed with each of the four female cancers.

Numbers of women who will develop cancer within the next 10 years…[1]

	per 1,000 women aged **45 years**	per 1,000 women aged **55 years**	per 1,000 women aged **65 years**	per 1,000 women aged **75 years**
Breast cancer	21	30	35	33
Ovarian cancer	2	3	4	4
Cervical cancer	2	2	1	1
Womb cancer	2	5	6	6

[1]All numbers refer to women from Germany; these are, however, comparable to the numbers of women from other European countries. German Centre for Cancer Registry Data within the Robert Koch Institute (2013). Database query: 1 August 2016, www.krebsdaten.de.

Women's cancer risk identification (WID) test

A test under development to identify a woman's risk of developing breast cancer, ovarian cancer, cervical cancer, and womb cancer

What are current approaches for healthy women to reduce the threat of these four female cancers?

Two approaches are currently used. The main approach – offered to all healthy women from a certain age on – is cancer screening such as mammography screening for breast cancer or ultrasound screening for ovarian cancer. Cancer screening is intended to detect cancer and precancerous lesions early. For the small proportion of women at very high risk another approach is preventive medication (e. g., with Tamoxifen©) and prophylactic surgery (e. g., removal of the breasts), which aim at preventing occurrence of cancer.

Currently used cancer screenings such as mammography can have benefits ***AND*** harms. The main benefit is defined as the number of women who will be saved from dying from cancer. The harms are defined by the number of women who experience false alarms, diagnoses of harmless forms of cancer (called overdiagnosis), and, as a consequence, unnecessary treatment (called overtreatment). The following table gives you the numbers for such a benefit-harm ratio for mammography. It shows that per 1,000 women aged 50 years or older who participated in mammography for 10 or more years, 1 woman would be saved from dying from breast cancer due to screening. At the same time, about 5 per 1,000 women who undergo screening would be wrongly diagnosed with progressive breast cancer and treated unnecessarily. Also, mammography has no effect on overall mortality from cancer.

Researchers assume that the benefit-harm ratio of screening would become more favourable if cancer screenings were tailored to a woman's personal risk of getting cancer. Particularly for women at higher-than-average risk, the benefits of screening may outweigh its harms, whereas for women at average or lower-than-average risk, less screening could be more advantageous in terms of avoiding the potential harms of screening.

Breast Cancer Early Detection

by Mammography

HARDING CENTER FOR RISK LITERACY

Mammography screening may reduce the number of women who die from breast cancer but this has no effect on overall cancer deaths. Among all women taking part in screening, some women will be overdiagnosed with non-progressive cancer and unnecessarily treated.

Numbers for women aged 50 years or older who did or did not participate in screening for about 10 years.

	1000 women without screening	1000 women with screening
**Benefits**		
How many women died from breast cancer?	5	4
How many women died from all types of cancer?	21	21
**Harms**		
How many women without cancer experienced false alarms or biopsies?	–	about 100
How many women with non-progressive cancer had unnecessary partial or complete breast removal?	–	5

Source: Gøtzsche, Jørgensen (2013). *Cochrane Database of Systematic Reviews* (6): CD001877.pub5
Numbers in the fact box are rounded. Where no data for women above 50 years of age are available, numbers refer to women above 40 years of age.
Date last updated: 13 March, 2014

Four cancers
One test

Women’s cancer risk identification (WID) test

A test under development to identify a woman’s risk of developing breast cancer, ovarian cancer, cervical cancer, and womb cancer

What is the rationale behind the currently developed WID test and what would it potentially change?

The rationale of the new WID test – currently under development – is to provide a woman with such a personalized risk prediction about getting certain female cancers **within the next 10 years.** The female cancers it targets are: breast cancer, ovarian cancer, cervical cancer, and womb cancer. In contrast to cancer screenings, the WID test is meant to predict the risk of these four female cancers ***before*** cancer is present. By using this test in the future, women may learn whether their personal risk is lower-than-average, at average, or higher-than-average.

To achieve this goal, the WID test will be based on the technology of *epigenomics.* Because each human being‘s *epigenome* records the life-time exposure to environmental cancer-triggering factors such as infections, diet, or smoking on an individual level, changes in our epigenome seem to be a promising target for predicting cancer risk. Not only may this technology assist in better predicting women’s personal cancer risk in the future, but it may also make more information available on the relation between a woman’s personal lifestyle and her cancer risk. The following figure illustrates the influence of some environmental factors on our epigenome.

If the WID test became available, it would be for women aged 50 years onwards. As the technology would use the epigenome of cervix cells, samples for the test would be obtained from cervical cells by a smear test. The test would be carried out by gynaecologists, general practitioners, and nurses who are qualified to perform smear tests. The test would take less than a minute and should not involve any pain. The cells taken from the smear test sample would be sent to a laboratory in order to test for your risk. When your results are made available, they would be discussed with your doctor at a follow-up meeting.

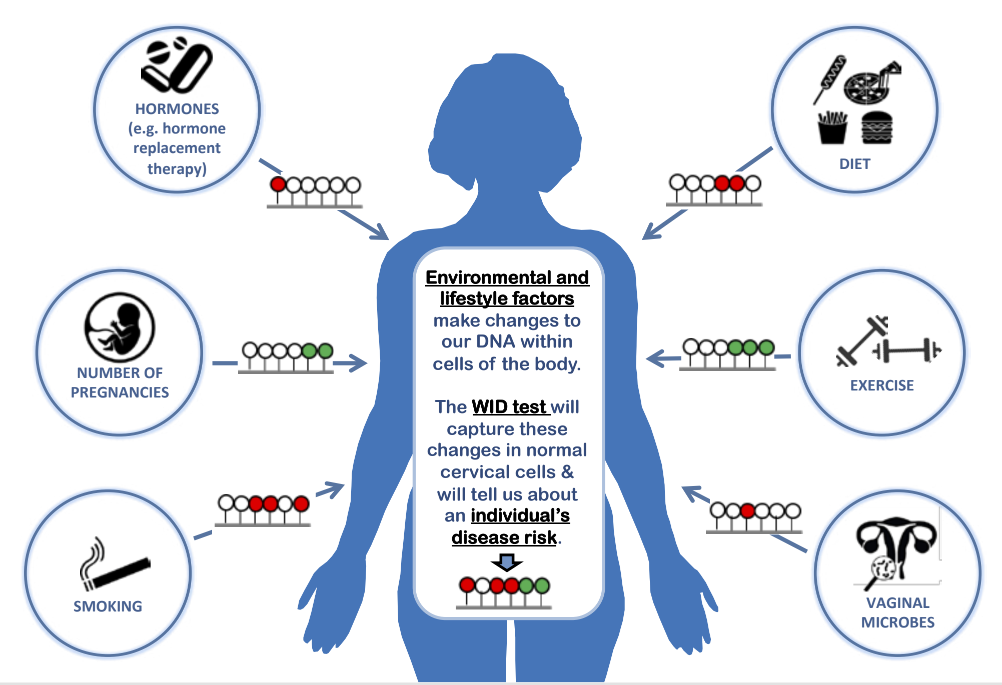

Figure: Various environmental factors trigger changes to our epigenome. The ‘lollipops‘ next to each factor represent examples of potential epigenome changes that can happen in response to that factor. In the example these changes can be good (green) or bad (red) and can vary in strength – the more lollipops marked, the stronger the change. The WID test can measure these changes and so be used to predict the risks of developing certain diseases.

Four cancers
One test

Women's cancer risk identification (WID) test

A test under development to identify a woman's risk of developing breast cancer, ovarian cancer, cervical cancer, and womb cancer

What are the potential benefits of taking the test?

Currently, the WID test is under clinical investigation in order to identify appropriate risk groups and interventions that work best for each risk group. If these risk groups and interventions are eventually identified, the WID risk test may help women and their doctors in the future adjust a woman's health care to her personal risks, with the following advantages:

- Women who are predicted to have a higher-than-average risk of developing any of the female cancers could take preventive actions (e. g., adopting a healthier lifestyle if risk is slightly elevated, preventive medication or prophylactic surgery if risk is high) in order to control or to lower their risk. Also, they could do more frequent screening to detect a cancer as early as possible.
- Women who are predicted to have an average or lower-than-average risk may decide to maintain their current lifestyle and have less screenings that entail potential harms.
- Because the WID test will be able to record changes in the epigenome that are caused by environmental and life-style factors, the test may also allow for monitoring how effective preventive actions such as changes in life-style are on reducing the personal cancer risk over time.

What are potential harms of taking the test?

No test is perfect. The WID test may lead to an inaccurate prediction of your risk of developing cancer and could therefore lead to unnecessary anxiety, diagnostic procedures, or medical interventions, or to the illusion of being at low risk when one is actually at higher risk. Even if the prediction is accurate, it does not mean that a woman with a higher-than-average risk will get cancer for certain and a woman with a lower-than-average risk will not get cancer.

Because numerical information about the quality of the test is not available at the current developmental stage, the likelihood of its making inaccurate predictions is not yet known. Furthermore, whether the WID test will eventually help in further reducing the number of women dying from any of the four female cancers or in reducing the number of women experiencing wrong diagnoses needs to be demonstrated in a larger clinical study in the future.

Who will be able to access my test data if I have the WID test in the future?

If you did not consent to the permanent storage of your test sample and results, your sample would be destroyed after risk estimation. As with other diagnostic and predictive tests, your risk information falls under medical confidentiality, meaning that access would be restricted to you, the laboratory, and your doctor.

Are there any other considerations?

The technology of the test makes information available on the relation between a woman's personal lifestyle and her cancer risk. Receiving the result of a higher-than-average risk may make you feel personally responsible for this result. In addition, many people often take predictive tests to reassure themselves that everything is fine, which is not always the case. Before having the test you should therefore also think about how you would cope with receiving a higher-than-average result. It is recommended that you talk to your doctor and your family members about your thoughts and concerns before having such a test.

4C forecee
Four cancers
One test

The FORECEE consortium with its following partners is responsible for the research and developmental work on the WID test

Who is responsible for the content of the leaflet?

Max Planck Institute for Human Development,
Harding Center for Risk Literacy
Lentzeallee 94
14195 Berlin (Germany)
Scientific project lead: O. Wegwarth / G. Gigerenzer
Contact: forecee@mpib-berlin.mpg.de

Where can you find more information about the test and the diseases?

www.forecee.eu
www.eveappeal.org.uk
www.cancerresearchuk.org

UCL
Karolinska Institutet
Erasmus MC University Medical Center Rotterdam
London School of Hygiene & Tropical Medicine
IEO Istituto Europeo di Oncologia
LMU Ludwig-Maximilians-Universität München
GATC Biotech
University Hospital Southampton NHS Foundation Trust
oncotyrol
Helse Bergen Haukeland University Hospital
EURAM
Harding Center for Risk Literacy
