# Supplementary material for: Do cancer risk and benefit–harm ratios influence women’s consideration of risk-reducing mastectomy? A scenario-based experiment in five European countries
Source: PLoS One. 2019 Jun 12;14(6):e0218188. doi: 10.1371/journal.pone.0218188 (PMC6561593; doi:10.1371/journal.pone.0218188)
Supplement: S2 Fig — (PDF) [file pone.0218188.s005.pdf]

For women at higher risk of getting breast cancer, undergoing prophylactic removal of the breasts (called mastectomy) is currently one medical intervention offered to reduce their risk of getting and potentially dying of breast cancer. Although the risk of getting breast cancer can be reduced by having a mastectomy, the surgical procedure itself entails potential harms.

Imagine you are at high risk and are offered the opportunity to undergo mastectomy. How large would the benefit need to be for you to accept the potential harms? Please go through the following ***hypothetic*** *scenarios, which show different odds for the benefit and harms. Please indicate for each scenario whether you would consider having prophylactic removal of your breasts in order to prevent the risk of dying of breast cancer or not.*
