# Supplementary material for: Do cancer risk and benefit–harm ratios influence women’s consideration of risk-reducing mastectomy? A scenario-based experiment in five European countries
Source: PLoS One. 2019 Jun 12;14(6):e0218188. doi: 10.1371/journal.pone.0218188 (PMC6561593; doi:10.1371/journal.pone.0218188)
Supplement: S3 Fig — (PDF) [file pone.0218188.s006.pdf]

Dotazník

Demografické údaje:

1) Kolik je vám let?
____

2) Jaké je vaše nejvyšší ukončené vzdělání?
- žádné ukončené vzdělání
- základní škola, bez maturity
- maturita nebo odpovídající zkouška
- vysoká škola

3) Byl vám někdy diagnostikován zhoubný nádor?
- ano
- ne
- nevím

  Pokud ano: jaký druh zhoubného nádoru:____________

4) Byl někdy diagnostikován jakýkoli druh zhoubného nádoru některému z členů vaší užší rodiny (např. rodičům, partnerovi, dětem)?
- ano
- ne
- nevím

  Pokud ano: jaký druh zhoubného nádoru:____________

Pochopení individuálního rizika zhoubného nádoru a časné diagnostiky

1) Představte si prosím 1 000 žen ve vašem věku *[zde automaticky uveďte věk co nejbližší věku respondentky]*.
U kolika z těchto 1 000 žen se během příštích 10 let vyvinou následující zhoubná onemocnění?

Rakovina prsu:	___ z 1 000 žen
Rakovina vaječníku:	___ z 1 000 žen
Rakovina děložního čípku:	___ z 1 000 žen
Rakovina dělohy:	___ z 1 000 žen

2) V níže uvedeném seznamu prosím zatrhněte tvrzení, které považujete za správné (správná je pouze jedna odpověď).

Preventivní vyšetření, jako je mamografie, ...
- má výhody, jako je snížená úmrtnost na rakovinu, a žádné nevýhody.
- má výhody, jako je snížená úmrtnost na rakovinu, a nevýhody, jako jsou zbytečné diagnózy a zbytečné léčení.
- pomáhá při předcházení rakovině, protože zjišťuje rakovinu dříve, než vznikne.

Obecné vnímání rizika

1) Jak pravděpodobné je podle vašeho soudu, že vám bude během příštích 10 let diagnostikována rakovina prsu, v porovnání s pravděpodobností, že vám bude během příštích 10 let diagnostikována osteoporóza?

x----------------x---------------x---------------x----------------x
1 2 3 4 5
(mnohem méně pravděpodobné) (stejně pravděpodobné) (mnohem pravděpodobnější)

2) Jak pravděpodobné je podle vašeho soudu, že vám bude během příštích 10 let diagnostikována rakovina prsu, v porovnání s pravděpodobností, že vám bude během příštích 10 let diagnostikována rakovina děložního čípku?

x----------------x---------------x---------------x----------------x
1 2 3 4 5
(mnohem méně pravděpodobné) (stejně pravděpodobné) (mnohem pravděpodobnější)

<u>Otázky o testu WID</u>

<u>Porozumění testům WID:</u>

1) Zatrhněte prosím všechny druhy zhoubného nádoru, na které je zaměřen test WID (správná je pouze jedna odpověď).

- rakovina vaječníku, rakovina dělohy, rakovina plic a rakovina slinivky břišní
- rakovina střev, rakovina prsu, rakovina děložního čípku a rakovina jater
- rakovina prsu, rakovina vaječníku, rakovina dělohy a rakovina děložního čípku

2) Co může pro ženu znamenat, když u ní výsledek testu říká, že riziko je u ní podprůměrné (správná je pouze jedna odpověď)?

- Pomocí několika preventivních vyšetření by u ní mohlo být sníženo riziko, že bude dostávat falešné poplašné zprávy a nepotřebnou léčbu.
- Pomocí vícerých preventivních vyšetření by se u ní mohlo snížit riziko úmrtí na rakovinu.
- Mohla by si být jista, že nikdy nedostane žádný z testovaných druhů rakoviny.

3) Co může pro ženu znamenat, když u ní výsledek testu říká, že riziko je u ní nadprůměrné (správná je pouze jedna odpověď)?

- Pomocí několika preventivních vyšetření by se u ní mohlo snížit riziko úmrtí na rakovinu.
- Pomocí vícerých preventivních vyšetření či preventivního podávání léků by se u ní mohlo snížit riziko úmrtí na rakovinu.
- Mohla by si být jista, že určitě dostane některý z testovaných druhů rakoviny.

4) Test WID má pomocí analýzy epigenomu předpovědět u ženy individuální riziko různých druhů rakoviny vyskytujících se u žen. Které z níže uvedených tvrzení o epigenomu je správné (správná je pouze jedna odpověď)?

- Životní prostředí a životní styl mění epigenom v buňkách.
- Epigenom se po celý život nemění.
- Epigenom je buňka s rakovinnými mutacemi.

Zaměření a účel testu WID

Test WID má předpovědět vaše individuální riziko onemocnění jedním nebo více druhy rakoviny vyskytujícími se u žen: rakoviny prsu, rakoviny vaječníku, rakoviny děložního čípku a rakoviny dělohy

1) Když uvažujete o testu WID, jak vnímáte poměr mezi výhodami a nevýhodami?

   x--------------------x------------------------x---------------------x----------------------x

1	2	3	4	5
Nevýhody převažují jasně výhody		Nevýhody a výhody jsou vyvážené		Výhody převažují jasně nevýhody

2) Mnohé ženy by rády věděly, jaké je riziko, že v budoucnu onemocní, jiné ženy to však vědět nechtějí. Když uvažujete o testu WID, chtěla byste vědět, jaké je riziko, že v průběhu příštích 10 let onemocníte jedním či více ze čtyř „ženských“ druhů rakoviny?

Rakovina prsu	O ano O ne
Rakovina dělohy	O ano O ne
Rakovina děložního čípku	O ano O ne
Rakovina vaječníku	O ano O ne

3) Kdyby byl test WID jednoduše proveditelný a byl k dispozici zdarma, nechala byste si jej provést, aby u vás bylo zjištěno riziko těchto čtyř druhů rakoviny?

   - Určitě bych si test NENECHALA udělat.
   - Pravděpodobně bych si test NENECHALA udělat.
   - Pravděpodobně bych si test nechala udělat.
   - Určitě bych si test nechala udělat.

4) V uplynulých měsících jsme se dotazovali různých skupin žen na jejich názory týkající se testů WID.

   V níže uvedeném seznamu jsou ukázány nejdůležitější důvody dotazovaných žen, které se vyslovily ***PRO*** provedení testu. Označte prosím číslem všechny důvody, které u vás osobně mluví ***pro*** test, a to podle pořadí důležitosti konkrétně pro vás. Začněte přitom číslem „1“ u nejzávažnějšího důvodu. V případě, že dva nebo více důvodů budou pro vás mít stejnou důležitost, zadejte prosím stejné číslo vícekrát. Pokud by uvedené důvody pro vás neměly žádnou důležitost, neuvádějte u nich žádné číslo.

Když si nechám test provést...

- budu mít menší obavy, že dostanu rakovinu.
- budu si v životě více uvědomovat, jaká mohu provádět opatření, například mít zdravější životní styl.
- budu mít lepší kontrolu nad svým životem (např. budu se rizikem zabývat již DNES, ještě než se rakovina objeví).
- budu při výběru strategických zdravotních opatření postupovat tak (např. individuálně přizpůsobenou frekvencí preventivních vyšetření), aby se co nejlépe předcházelo onemocnění rakovinou nebo úmrtí na rakovinu.
- budu se snažit o včasné provádění strategií k potlačení nadprůměrného rizika.

V níže uvedeném seznamu jsou ukázány nejdůležitější důvody u dotazovaných žen, které se vyslovily ***PROTI*** provedení testu. Označte prosím číslem všechny důvody, které u vás osobně mluví ***proti*** testu, a to podle pořadí důležitosti pro vás. Začněte přitom číslem „1“ u nejzávažnějšího důvodu. V případě, že dva nebo více důvodů budou pro vás mít stejnou důležitost, zadejte prosím stejné číslo vícekrát. Pokud by uvedené důvody pro vás neměly žádnou důležitost, neuvádějte u nich žádné číslo.

Provedení testu...

- bych považovala za bezcenné, protože výsledek testu je pouze odhadnutá hodnota nic neříkající o tom, zda rakovinu opravdu dostanu.
- by u mne v případě nadprůměrného rizika vedlo k pocitu, že jsem vinná nebo odpovědná za výsledek, protože existuje souvislost mezi výsledkem testu a mým životním stylem.
- by mne i mou rodinu v případě nadprůměrného rizika zbytečně zneklidnilo a ovlivnilo by negativně mou dnešní kvalitu života.
- by mě v případě nadprůměrného rizika dostalo pod tlak, abych změnila svůj životní styl na zdravější, nebo abych také abych si častěji nechala provádět preventivní vyšetření.
- by v případě nadprůměrného rizika způsobilo, že bych pořád musela poslouchat o rakovině.

5) *[Na obrazovce se automaticky zobrazí zatrhnuté důvody]* Zde vidíte důvody, které jste vybrala ***PRO*** provedení testů WID. Když se na ně podíváte, je mezi nimi JEDEN důvod, který je tak silný, že by převážil všechny ostatní důvody?

   _ Ano

      - Pokud ano, rozhodující důvod je: _________

   _ Ne, přihlédla bych určitě ke všem důvodům, které jsem v seznamu zvolila.

6) *Na obrazovce se automaticky zobrazí zaškrtnuté důvody]* Zde vidíte důvody, které jste vybrala ***PROTI*** provedení testů WID. Když se na ně podíváte, je mezi nimi JEDEN důvod, který je tak silný, že by převážil všechny ostatní důvody?

   _ Ano

- Pokud ano, rozhodující důvod je: __________

_ Ne, přihlédla bych určitě ke všem důvodům, které jsem v seznamu zvolila.

Zhodnocení poměru výhod a nevýhod:

Ženy s vyšším rizikem rakoviny prsu si mohou nechat preventivně prsy odstranit (mastektomie), aby se snížilo riziko, že onemocní rakovinou prsu a zemřou na ni. I když riziko onemocnění a úmrtí na rakovinu prsu lze mastektomií snížit, má tento chirurgický výkon samozřejmě i možné nevýhody.

Představte si, že je u vás nadprůměrné riziko a nabízí se vám možnost mastektomie. Jak velké by musely být výhody, aby převážily potenciální nevýhody? Projděte si prosím následující ***hypotetické*** možnosti, které ukazují různý poměr výhod a nevýhod. Uveďte prosím u každé možnosti, zda byste u ní zvažovala odnětí prsou či nikoli.

	Z 1 000 žen, jako jste vy, které si prsy preventivně odstranit nenechají	Z 1 000 žen, jako jste vy, které si prsy preventivně odstranit nechají
Výhody:		
Počet žen, které během příštích 10 let zemřou na rakovinu prsu	**10**	**2**
Nevýhody:		
Závažné komplikace během operace a po operaci (např. infekce, špatné hojení rány)	**-**	**100**

Uvažovala byste o preventivním odstranění prsů?

O ano O ne

	Z 1 000 žen, jako jste vy, které si prsy preventivně odstranit <u>nenechají</u>	Z 1 000 žen, jako jste vy, které si prsy preventivně odstranit nechají
Výhody:		
Počet žen, které během příštích 10 let zemřou na rakovinu prsu	**10**	**6**
Nevýhody:		
Závažné komplikace během operace a po operaci (např. infekce, špatné hojení rány)	**-**	**100**

Uvažovala byste o preventivním odstranění prsů?

O ano O ne

	Z 1 000 žen, jako jste vy, které si prsy preventivně odstranit nenechají	Z 1 000 žen, jako jste vy, které si prsy preventivně odstranit nechají
Výhody:		
Počet žen, které během příštích 10 let zemřou na rakovinu prsu	**5**	**3**
Nevýhody:		
Závažné komplikace během operace a po operaci (např. infekce, špatné hojení rány)	**-**	**100**

Uvažovala byste o preventivním odstranění prsů?

O ano O ne

	Z 1 000 žen, jako jste vy, které si prsy preventivně odstranit <u>nenechají</u>	Z 1 000 žen, jako jste vy, které si prsy preventivně odstranit nechají
Výhody:		
Počet žen, které během příštích 10 let zemřou na rakovinu prsu	**5**	**1**
Nevýhody:		
Závažné komplikace během operace a po operaci (např. infekce, špatné hojení rány)	**-**	**100**

Uvažovala byste o preventivním odstranění prsů?

O ano O ne

	Z 1 000 žen, jako jste vy, které si prsy preventivně odstranit nenechají	Z 1 000 žen, jako jste vy, které si prsy preventivně odstranit nechají
Výhody:		
Počet žen, které během příštích 10 let zemřou na rakovinu prsu	**20**	**12**
Nevýhody:		
Závažné komplikace během operace a po operaci (např. infekce, špatné hojení rány)	**-**	**100**

Uvažovala byste o preventivním odstranění prsů?

O ano O ne

	Z 1 000 žen, jako jste vy, které si prsy preventivně odstranit nenechají	Z 1 000 žen, jako jste vy, které si prsy preventivně odstranit nechají
Výhody:		
Počet žen, které během příštích 10 let zemřou na rakovinu prsu	**20**	**4**
Nevýhody:		
Závažné komplikace během operace a po operaci (např. infekce, špatné hojení rány)	**-**	**100**

Uvažovala byste o preventivním odstranění prsů?

O ano O ne

[Debriefing]

Všechny možnosti, které jste právě zvažovala, a příslušné výhody a nevýhody mastektomie byly hypotetické.

Počty ukázané u jednotlivých možností a příslušná rizika úmrtí na rakovinu prsu a také u výhod a nevýhod mastektomie neodpovídají skutečným počtům z klinicky doložitelných studií.

U těchto možností jsme systematicky měnili počty u rizika úmrtí na rakovinu prsu bez mastektomie a také u výhod a nevýhod mastektomie. Naším cílem přitom bylo usnadnit pochopení, jaký musí být u mastektomie poměr výhod a nevýhod, aby se žena rozhodla nechat si tuto operaci provést.

Další informace naleznete mimo jiné zde:
www.forecee.eu
www.eveappeal.org.uk
www.cancerresearchuk.org.
www.rki.de

Děkujeme vám za účast v naší studii.

Max-Planck-Institut für Bildungsforschung, Harding-Zentrum für Risikokompetenz

Lentzeallee 94

14195 Berlín

Vědecké vedení projektu: O. Wegwarth / G. Gigerenzer

Kontakt: forecee@mpib-berlin.mpg.de
