# Supplementary material for: Do cancer risk and benefit–harm ratios influence women’s consideration of risk-reducing mastectomy? A scenario-based experiment in five European countries
Source: PLoS One. 2019 Jun 12;14(6):e0218188. doi: 10.1371/journal.pone.0218188 (PMC6561593; doi:10.1371/journal.pone.0218188)
Supplement: S4 Fig — (PDF) [file pone.0218188.s007.pdf]

Fragebogen

Demografie:

1) Wie alt sind Sie?
____

2) Welches ist Ihr höchster Bildungsabschluss?
- kein Schulabschluss
- einfacher Schulabschluss ohne Abitur
- Abitur oder Äquivalent
- Hochschulabschluss

3) Wurde bei Ihnen jemals Krebs diagnostiziert?
- ja
- nein
- ich weiß es nicht
  Falls ja: welche Art von Krebs:____________

4) Wurde jemals bei einem der Mitglieder Ihrer engeren Familie (z.B. Eltern, Partner, Kinder) irgendeine Art von Krebs diagnostiziert?
- ja
- nein
- ich weiß es nicht
  Falls ja: welche Art von Krebs:____________

Persönliches Krebsrisiko und Früherkennung verstehen

1) Bitte stellen Sie sich 1.000 Frauen in Ihrem Alter vor *[Include age that is closest to the age of respondent automatically here]*.
Wie viele von diesen 1.000 Frauen werden innerhalb der nächsten 10 Jahre die folgenden Krebserkrankungen entwickeln?

Brustkrebs:	___ von 1.000 Frauen
Eierstockkrebs:	___ von 1.000 Frauen
Gebärmutterhalskrebs:	___ von 1.000 Frauen
Gebärmutterkrebs:	___ von 1.000 Frauen

2) Bitte kreuzen Sie die Aussage in der folgenden Liste an, von der Sie meinen, dass sie richtig ist (nur eine Antwort ist richtig).

Früherkennungen wie die Mammographie...
- bringen Nutzen wie eine verringerte Krebssterblichkeit, aber keinen Schaden.
- bringen Nutzen wie eine verringerte Krebssterblichkeit und Schaden wie unnötige Diagnosen und unnötige Behandlungen.
- helfen Krebs vorzubeugen, weil sie Krebs entdecken bevor dieser entsteht.

Allgemeine Risikowahrnehmung

1) Im Vergleich zur Wahrscheinlichkeit eine Osteoporose-Diagnose in den nächsten 10 Jahren zu erhalten, wie wahrscheinlich ist es Ihrer Meinung nach, innerhalb der nächsten 10 Jahre eine Brustkrebs-Diagnose zu erhalten?

x----------------x---------------x---------------x----------------x
1 2 3 4 5
(Viel weniger wahrscheinlich) (gleich wahrscheinlich) (Sehr viel wahrscheinlicher)

2) Im Vergleich zur Wahrscheinlichkeit eine Gebärmutterhalskrebs-Diagnose in den nächsten 10 Jahren zu erhalten, wie wahrscheinlich ist es Ihrer Meinung nach, innerhalb der nächsten 10 Jahre eine Brustkrebs-Diagnose zu erhalten?

x----------------x---------------x---------------x----------------x
1 2 3 4 5
(Viel weniger wahrscheinlich) (gleich wahrscheinlich) (Sehr viel wahrscheinlicher)

Fragen zum WID-Test

Verständnis des WID-Tests:

1) Bitte kreuzen Sie jene Krebsarten an, auf die der WID-Test abzielt (nur eine Antwort ist richtig).

- Eierstockkrebs, Gebärmutterkrebs, Lungenkrebs und Bauchspeicheldrüsenkrebs
- Darmkrebs, Brustkrebs, Gebärmutterhalskrebs und Leberkrebs
- Brustkrebs, Eierstockkrebs, Gebärmutterkrebs und Gebärmutterhalskrebs

2) Was könnte eine Frau mit einem Testergebnis anfangen, welches aussagt, dass sie ein unterdurchschnittliches Risiko hat (nur eine Antwort ist richtig)?

- Sie könnte durch weniger Früherkennungen ihr Risiko verringern, Fehlalarme und unnötige Behandlungen zu erhalten.
- Sie könnte durch mehr Früherkennungen ihr Risiko verringern, an Krebs zu versterben.
- Sie könnte sich sicher sein, dass sie definitiv keine der getesteten Krebsarten bekommen wird.

3) Was könnte eine Frau mit einem Testergebnis anfangen, welches aussagt, dass sie ein überdurchschnittliches Risiko hat (nur eine Antwort ist richtig)?

- Sie könnte durch weniger Früherkennungen ihr Risiko verringern, an Krebs zu versterben.
- Sie könnte durch mehr Früherkennungen oder präventive Medikamenteneinnahme ihr Risiko verringern, an Krebs zu versterben.
- Sie könnte sich sicher sein, dass sie definitiv eine der getesteten Krebsarten bekommen wird.

4) Der WID-Test soll einer Frau das persönliche Risiko für verschiedene weibliche Krebsarten vorhersagen, indem er ihr Epigenom analysiert. Welche der folgenden Aussagen zum Epigenom ist korrekt (nur eine Antwort ist richtig)?

- Ihre Umwelt und Ihr Lebensstil verändern das Epigenom Ihrer Zellen.
- Das Epigenom bleibt über die gesamte Lebensspanne unverändert.
- Das Epigenom ist eine Zelle mit Krebsmutationen.

Einstellungen und Intentionen im Hinblick auf den WID-Test

Der WID-Test soll Ihr persönliches Risiko für eine oder auch mehrere der vier weiblichen Krebsarten vorhersagen: Brustkrebs, Eierstockkrebs, Gebärmutterhalskrebs, Gebärmutterkrebs

1) Wenn Sie über den WID-Test nachdenken, wie fühlt sich für Sie das Verhältnis zwischen Nutzen und Schaden an?

x--------------------x------------------------x---------------------x----------------------x

1	2	3	4	5
Der Schaden überwiegt klar den Nutzen		Schaden und Nutzen sind ausgeglichen		Der Nutzen überwiegt klar den Schaden

2) Während manche Frauen ihr Risiko, in Zukunft zu erkranken, gerne wissen würden, möchten andere Frauen das nicht wissen. Wenn Sie an den WID-Test denken, möchten Sie ihr Risiko wissen, an einer oder mehreren der vier weiblichen Krebsarten innerhalb der nächsten 10 Jahre zu erkranken?

Für Brustkrebs	O ja O nein
Für Gebärmutterkrebs	O ja O nein
Für Gebärmutterhalskrebs	O ja O nein
Für Eierstockkrebs	O ja O nein

3) Wenn der WID-Test gegenwärtig schon einfach durchführbar und frei verfügbar wäre, würden Sie diesen durchführen lassen, um Ihr Risiko für die vier Krebsarten bestimmen zu lassen?

- Ich würde den Test sicher NICHT machen.
- Ich würde den Test wahrscheinlich NICHT machen.
- Ich würde den Test wahrscheinlich machen
- Ich würde den Test sicher machen.

4) In den letzten Monaten haben wir verschiedene Gruppen von Frauen zu ihren Gedanken bezüglich des WID-Tests befragt.

Die folgende Liste zeigt die wichtigsten Gründe der befragten Frauen, die ***FÜR*** eine Teilnahme an dem Test sprachen. Bitte geben Sie allen Gründen, die auch für Sie persönlich ***für*** den Test sprechen würden, eine Nummer in der Reihenfolge der Bedeutung für Sie. Starten Sie dabei bitte mit einer „1" für den wichtigsten Grund. Für den Fall, dass zwei oder mehr Gründe gleichbedeutend für Sie sind,

vergeben Sie bitte dieselbe Zahl mehrfach. Sollte einer der gelisteten Gründe keine Bedeutung für Sie haben, geben Sie diesem bitte keine Nummer.

Den Test durchführen zu lassen...

- würde meine Sorge reduzieren, Krebs zu bekommen.
- ließe mich mein Leben bewusster leben, indem ich Maßnahmen wie zum Beispiel einen gesünderen Lebensstil ergreifen würde.
- würde die wahrgenommene Kontrolle über mein Leben erhöhen (z.B. schon HEUTE mit dem Risiko auseinandersetzen, bevor der Krebs entsteht).
- würde mich bei der Auswahl meiner medizinischen Versorgungsstrategie unterstützen (z.B. persönlich zugeschnittene Häufigkeit von Früherkennungen), um bestmöglich Krebs oder dem Krebstod vorzubeugen.
- würde mir helfen, frühzeitig Strategien zur Bewältigung eines überdurchschnittlichen Risikos zu entwickeln.

Die folgende Liste zeigt die wichtigsten Gründe der befragten Frauen, die ***GEGEN*** eine Teilnahme an dem Test sprachen. Bitte geben Sie allen Gründen, die auch für Sie persönlich ***gegen*** den Test sprechen würden, eine Nummer in der Reihenfolge der Bedeutung für Sie. Starten Sie dabei bitte mit einer „1“ für den wichtigsten Grund. Für den Fall, dass zwei oder mehr Gründe gleichbedeutend für Sie sind, vergeben Sie bitte dieselbe Zahl mehrfach. Sollte einer der gelisteten Gründe keine Bedeutung für Sie haben, geben Sie diesem bitte keine Nummer.

Den Test zu machen...

- würde ich als sinnlos erachten, weil das Testergebnis nur ein Schätzwert ist, der mir nichts darüber sagt, ob ich wirklich Krebs bekommen werde.
- würde mir im Falle eines überdurchschnittlichen Risikos das Gefühl geben, schuldig oder verantwortlich für das Ergebnis zu sein, da es die Verbindung zwischen dem Testergebnis und meinem Lebensstil in der Vergangenheit gibt.
- würde meine Familie und mich im Falle eines überdurchschnittlichen Risikos unnötig beunruhigen und meine heutige Lebensqualität negativ beeinflussen.
- würde mich im Falle eines überdurchschnittlichen Risikos unter Druck setzen, meinen Lebensstil gesundheitsbezogen anzupassen oder auch mehr Krebsfrüherkennungen machen zu lassen.
- würde mich im Fall eines überdurchschnittlichen Risikos ständig nach dem Krebs horchen lassen.

5) *[Screen presents ticked reasons automatically]* Hier sehen Sie die Gründe, welche Sie ***FÜR*** die Durchführung des WID-Tests auswählten. Wenn Sie sich diese anschauen, gibt es EINEN Grund, der so stark ist, dass er alle anderen Gründe überwiegt?

   _ Ja

      - Falls ja, der entscheidende Grund ist: _________

   _ Nein, ich würde definitiv alle Gründe, die ich auf meiner Liste auswählte, berücksichtigen.

6) *Screen presents ticked reasons automatically]* Hier sehen Sie die Gründe, welche Sie ***GEGEN*** die Durchführung des WID-Tests auswählten. Wenn Sie sich diese anschauen, gibt es EINEN Grund, der so stark ist, dass er alle anderen Gründe überwiegt?

_ Ja

- Falls ja, der entscheidende Grund ist: __________

_ Nein, ich würde definitiv alle Gründe, die ich auf meiner Liste auswählte, berücksichtigen.

Bewertung des Nutzen-Schaden-Verhältnisses:

Frauen mit höherem Brustkrebsrisiko können sich vorsorglich ihre Brüste abnehmen lassen (Mastektomie), um das Risiko zu senken an Brustkrebs zu erkranken und zu sterben. Obwohl das Risiko an Brustkrebs zu erkranken oder zu sterben durch die Mastektomie herabgesetzt werden kann, birgt dieser chirurgische Eingriff selbst natürlich auch potenzielle Schäden.

Stellen Sie sich vor, Sie haben ein überdurchschnittliches Krebsrisiko und es bietet sich Ihnen die Möglichkeit zur Mastektomie. Wie groß müsste der Nutzen für Sie sein, um die potenziellen Schäden aufzuwiegen? Bitte gehen Sie durch die folgenden ***hypothetischen*** Szenarien, die verschiedene Nutzen-Schaden-Verhältnisse zeigen. Geben Sie bitte für jedes Szenario an, ob Sie eine vorsorgliche Abnahme Ihrer Brüste in Erwägung ziehen würden oder nicht.

[item manipulation plan]

Basisrisiko	Relative Risikoreduktion -40%	Relative Risikoreduktion -80%
5 / 1000	3 / 1000	1 / 1000
10 / 1000	6 / 1000	2 / 1000
20 / 1000	12 / 1000	4 / 1000

[Die nachfolgenden Tabellen=Items werden den Teilnehmerinnen in zufälliger Reihenfolge gezeigt]

	Von je 1.000 Frauen wie Sie, die sich <u>nicht</u> vorsorglich die Brüste abnehmen lassen	Von je 1.000 Frauen wie Sie, die sich vorsorglich die Brüste abnehmen lassen
Nutzen:		
Anzahl Frauen, die in den nächsten 10 Jahren an Brustkrebs sterben würden	**10**	**2**
Schäden:		
Schwere Komplikationen während und nach der Operation (z.B. Infektion, schlechte Wundheilung)	**-**	**100**

Würden Sie in Betracht ziehen, Ihre Brüste vorsorglich abnehmen zu lassen?

O ja O nein

	Von je 1.000 Frauen wie Sie, die sich <u>nicht</u> vorsorglich die Brüste abnehmen lassen	Von je 1.000 Frauen wie Sie, die sich vorsorglich die Brüste abnehmen lassen
Nutzen:		
Anzahl Frauen, die in den nächsten 10 Jahren an Brustkrebs sterben würden	**10**	**6**
Schäden:		
Schwere Komplikationen während und nach der Operation (z.B. Infektion, schlechte Wundheilung)	**-**	**100**

Würden Sie in Betracht ziehen, Ihre Brüste vorsorglich abnehmen zu lassen?

O ja O nein

	Von je 1.000 Frauen wie Sie, die sich nicht vorsorglich die Brüste abnehmen lassen	Von je 1.000 Frauen wie Sie, die sich vorsorglich die Brüste abnehmen lassen
Nutzen:		
Anzahl Frauen, die in den nächsten 10 Jahren an Brustkrebs sterben würden	**5**	**3**
Schäden:		
Schwere Komplikationen während und nach der Operation (z.B. Infektion, schlechte Wundheilung)	**-**	**100**

Würden Sie in Betracht ziehen, Ihre Brüste vorsorglich abnehmen zu lassen?

O ja O nein

	Von je 1.000 Frauen wie Sie, die sich nicht vorsorglich die Brüste abnehmen lassen	Von je 1.000 Frauen wie Sie, die sich vorsorglich die Brüste abnehmen lassen
Nutzen:		
Anzahl Frauen, die in den nächsten 10 Jahren an Brustkrebs sterben würden	**5**	**1**
Schäden:		
Schwere Komplikationen während und nach der Operation (z.B. Infektion, schlechte Wundheilung)	**-**	**100**

Würden Sie in Betracht ziehen, Ihre Brüste vorsorglich abnehmen zu lassen?

O ja O nein

	Von je 1.000 Frauen wie Sie, die sich nicht vorsorglich die Brüste abnehmen lassen	Von je 1.000 Frauen wie Sie, die sich vorsorglich die Brüste abnehmen lassen
Nutzen:		
Anzahl Frauen, die in den nächsten 10 Jahren an Brustkrebs sterben würden	**20**	**12**
Schäden:		
Schwere Komplikationen während und nach der Operation (z.B. Infektion, schlechte Wundheilung)	**-**	**100**

Würden Sie in Betracht ziehen, Ihre Brüste vorsorglich abnehmen zu lassen?

O ja O nein

	Von je 1.000 Frauen wie Sie, die sich nicht vorsorglich die Brüste abnehmen lassen	Von je 1.000 Frauen wie Sie, die sich vorsorglich die Brüste abnehmen lassen
Nutzen:		
Anzahl Frauen, die in den nächsten 10 Jahren an Brustkrebs sterben würden	**20**	**4**
Schäden:		
Schwere Komplikationen während und nach der Operation (z.B. Infektion, schlechte Wundheilung)	**-**	**100**

Würden Sie in Betracht ziehen, Ihre Brüste vorsorglich abnehmen zu lassen?

O ja O nein

[Debriefing]

Alle von Ihnen soeben bearbeiteten Szenarien zum Nutzen und Schaden einer Mastektomie waren hypothetisch.

Die in den Szenarien gezeigten Zahlen zum Risiko eines Krebstodes ohne Mastektomie sowie zum Nutzen und Schaden der Mastektomie korrespondieren nicht mit realen Zahlen aus klinisch belastbaren Studien.

Wir veränderten in den Szenarien systematisch die Zahlen zum Krebstodrisiko ohne Mastektomie sowie zum Nutzen und Schaden der Mastektomie wie in der nachfolgenden Tabelle dargestellt. Unser Ziel war es dabei, besser zu verstehen, in welchem Verhältnis der Nutzen zum Schaden der Mastektomie stehen muss, damit Frauen bereit wären, diesen chirurgischen Eingriff durchführen zu lassen.

Krebstod-Risiko ohne Mastektomie	Krebstod-Risiko mit Mastektomie (schwache Risikoreduktion)	Krebstod-Risiko mit Mastektomie (starke Risikoreduktion)
5 / 1000	3 / 1000	1 / 1000
10 / 1000	6 / 1000	2 / 1000
20 / 1000	12 / 1000	4 / 1000

Wenn Sie weitere Informationen benötigen, finden Sie diese u.a. unter:
www.forecee.eu
www.eveappeal.org.uk
www.cancerresearchuk.org.
www.rki.de

Wir danken Ihnen für Ihre Teilnahme an unserer Studie.

Max-Planck-Institut für Bildungsforschung, Harding-Zentrum für Risikokompetenz

Lentzeallee 94

14195 Berlin

Wissenschaftliche Projektleitung: O. Wegwarth / G. Gigerenzer

Kontakt: forecee@mpib-berlin.mpg.de
