# Supplementary material for: Do cancer risk and benefit–harm ratios influence women’s consideration of risk-reducing mastectomy? A scenario-based experiment in five European countries
Source: PLoS One. 2019 Jun 12;14(6):e0218188. doi: 10.1371/journal.pone.0218188 (PMC6561593; doi:10.1371/journal.pone.0218188)
Supplement: S5 Fig — (PDF) [file pone.0218188.s008.pdf]

Questionnaire for the leaflet on the WID test

Demographics:

1) How old are you?
____

2) What is your highest formal degree of education?
- No general certificate of secondary education
- General Certificate of Secondary Education (GCSE)
- General Certificate of Education Advanced Level (A-Level)
- University Degree

3) Have you ever had a cancer diagnosis?
- yes
- no
- I don't know
  If yes: What type of cancer?____________

4) Has a member of your close family (e.g., parents, spouse, children) ever had any type of cancer?
- yes
- no
- I don't know
  If yes: What type of cancer?____________

Understanding personal cancer risk and screening:

1) Please imagine 1,000 women at the age of xx, *[include age that is closest to the age of respondent automatically here]*.
How many women of these 1,000 would develop the following cancers within the next 10 years?

Breast cancer: ___ out of 1,000 women
Ovarian cancer: ___ out of 1,000 women
Cervical cancer: ___ out of 1,000 women
Womb cancer: ___ out of 1,000 women

2) Please tick that statement in the list that you think is correct (one answer only).

Having basic cancer screening tests such as mammography....
- comes with benefits such as a reduction of deaths from cancer but no harms
- comes with the benefit of reducing deaths from cancer and with harms such as unnecessary diagnoses and treatments
- helps prevent cancer because it finds cancer before it starts

General risk perception:

1) Compared to the risk of being diagnosed with osteoporosis, how likely do you think is it to be diagnosed with breast cancer within the next 10 years?

x----------------x--------------x--------------x---------------x
1 2 3 4 5
(Much less likely) (Equally likely) (Much more likely)

2) Compared with the risk of being diagnosed with cervical cancer, how likely do you think is it to be diagnosed with breast cancer within the next 10 years?

x----------------x--------------x--------------x---------------x
1 2 3 4 5
(Much less likely) (Equally likely) (Much more likely)

<u>Questions about the WID test</u>
<u>Understanding the WID test:</u>

1) Please tick the types of cancer that the WID test targets. (One answer only).

- ovarian cancer, womb cancer, lung cancer, and pancreatic cancer
- colorectal cancer, breast cancer, cervical cancer, and liver cancer
- breast cancer, ovarian cancer, womb cancer, and cervical cancer

2) What can a woman do with a test result that tells her that she has a lower-than-average risk? (One answer only)

- Reduce her risk of getting false alarms and unnecessary treatment by taking less intensive screening.
- Reduce her risk of dying of cancer by taking more intensive screening
- Have the certainty that she will definitely not get one of the tested types of cancer.

3) What could a woman do with a test result that tells her that she is at higher-than-average risk? (One answer only).

- Reduce her risk of dying of cancer by taking less intensive screening
- Reduce her risk of dying of cancer by taking more intensive screening or preventive medication.
- Have the certainty that she will definitely get one of the tested types of cancer

4) The WID test aims at predicting a woman's risk of developing female cancers by analysing her epigenome. Which of the following statements about the epigenome is correct (please tick only one answer):

- Your environment and lifestyle is changing the epigenome of your cells
- The epigenome always stays the same during your lifetime
- The epigenome is a cell with cancerous mutations.

Attitude and intentions respecting the WID test:

The WID test is meant to predict your personal risks of getting one or more of the four female cancers: breast cancer, ovarian cancer, cervical cancer, womb cancer.

1) When thinking of the WID test, how do you feel about the balance between benefits and harms?

x--------------------x------------------------x---------------------x----------------------x

1	2	3	4	5
The harms clearly outweigh the benefits		The harms and benefits are balanced		The benefits clearly outweigh the harms

2) Whereas some women prefer to know their risks of getting a disease in the future, others prefer not to. Thinking of the WID test, do you want to know your risk of developing one or more of the following four female cancers within the next 10 years?

For breast cancer	O yes O no
For womb cancer	O yes O no
For cervical cancer	O yes O no
For ovarian cancer	O yes O no

3) If already easily and freely available now, would you do the WID test to predict your risk of developing any of the four female cancers?

- I would **definitely NOT** do the test.
- I would **probably NOT** do the test.
- I would **probably do** the test.
- I would **definitely do** the test.

4) In the past months we have interviewed several groups of women about their thoughts concerning the WID test.

The following list presents the main reasons of the interviewed women **in favour of** doing the WID test. Please tick all reasons that matter for you personally and number them in order of their importance to you. Please start with "1" for the most important reason. In case of an equal importance of two or more reasons you can give reasons the same number. In case one or more reasons do not matter for you, please do not tick or number it.

Having the test …

- would reduce my concerns about getting cancer.
- would make me live my life more mindfully by doing things such as adopting a healthier lifestyle.
- would increase my perceived control of my life (e.g., to already discuss the risk of being potentially diagnosed with the disease before it develops).

- would help guide my medical strategy (e.g., individualise my screening uptake) to best prevent cancer or cancer death.
- would help me develop coping strategies early on in case of higher-than-average risk.

The following list presents the main reasons of the interviewed women **against** doing the WID test. Please tick all reasons that matter to you personally and number them in order of their importance to you. Please start with "1" for the most important reason. In case of an equal importance of two or more reasons you can give reasons the same number. In case one or more reasons do not matter for you, please do not tick or number them.

Having the test …

- would be of no use to me because the test result is only an estimate that tells me nothing about whether I will be getting cancer for sure.
- may make me feel guilty or responsible, if I am shown to be at higher-than-average risk, because of the connection between this result and my lifestyle in the past.
- would unnecessarily worry me and my family and negatively affect my quality of life if I am shown to be at higher-than-average risk.
- may put pressure on me to adopt a lifestyle related to my risk or have more cancer screening tests in case I am at higher-than-average risk.
- would make me expect the onset of cancer permanently if I am at higher-than-average risk.

5) *[Screen presents ticked reasons automatically]* Here are the reasons you chose in ***favour of*** having the WID test. Looking at them, is there ONE reason that is so strong that it would outweigh all the other reasons for having the WID risk test?

_ Yes
- If yes, the decisive reason is: _________
_ No, I would definitely consider all of the reasons I ticked on the list.

6) *[Screen presents ticked reasons automatically]* Here are the reasons you chose ***against*** having the WID test. Looking at them, is there ONE reason that is so strong that it would outweigh all the other reasons for not having the WID risk test?

_ Yes
- If yes, the decisive reason is: _________
_ No, I would definitely consider all of the reasons I ticked on the list.

Evaluation of benefit-harm ratio

For women at higher risk of getting breast cancer, undergoing prophylactic removal of the breasts (called mastectomy) is currently one medical intervention offered to reduce their risk of getting and potentially dying of breast cancer. Although the risk of getting breast cancer can be reduced by having a mastectomy, the surgical procedure itself entails potential harms.

Imagine you are at high risk and are offered the opportunity to undergo mastectomy. How large would the benefit need to be for you to accept the potential harms? Please go through the following ***hypothetic*** scenarios, which show different odds for the benefit and harms. Please indicate for each scenario whether you would consider having prophylactic removal of your breasts in order to prevent the risk of dying of breast cancer or not.

[item manipulation plan]

Baseline risk	RR -40%	RR -80%
5 / 1000	3 / 1000	1 / 1000
10 / 1000	6 / 1000	2 / 1000
20 / 1000	12 / 1000	4 / 1000

[Tables=items are presented to participants in random order]

	Out of 1,000 women like you who do not undergo prophylactic removal of the breasts	Out of 1,000 women like you who undergo prophylactic removal of the breasts
Benefits:		
Number of women who would die of breast cancer within 10 years	**10**	**6**
Harms:		
Severe complications during and following the surgery (e.g., infection, impaired healing)	**-**	**100**

Would you consider having prophylactic removal of your breasts?

O yes O no

	Out of 1,000 women like you who do not undergo prophylactic removal of the breasts	Out of 1,000 women like you who undergo prophylactic removal of the breasts
Benefits:		
Number of women who would die of breast cancer within 10 years	**10**	**2**
Harms:		
Severe complications during and following the surgery (e.g., infection, impaired healing)	**-**	**100**

Would you consider having prophylactic removal of your breasts?

O yes O no

	Out of 1,000 women like you who do not undergo prophylactic removal of the breasts	Out of 1,000 women like you who undergo prophylactic removal of the breasts
Benefits:		
Number of women who would die of breast cancer within 10 years	**5**	**3**
Harms:		
Severe complications during and following the surgery (e.g., infection, impaired healing)	**-**	**100**

Would you consider having prophylactic removal of your breasts?

O yes O no

	Out of 1,000 women like you who do not undergo prophylactic removal of the breasts	Out of 1,000 women like you who undergo prophylactic removal of the breasts
Benefits:		
Number of women who would die of breast cancer within 10 years	**5**	**1**
Harms:		
Severe complications during and following the surgery (e.g., infection, impaired healing)	**-**	**100**

Would you consider having prophylactic removal of your breasts?

O yes O no

	Out of 1,000 women like you who do not undergo prophylactic removal of the breasts	Out of 1,000 women like you who undergo prophylactic removal of the breasts
Benefits:		
Number of women who would die of breast cancer within 10 years	**20**	**12**
Harms:		
Severe complications during and following the surgery (e.g., infection, impaired healing)	**-**	**100**

Would you consider having prophylactic removal of your breasts?

O yes O no

	Out of 1,000 women like you who do not undergo prophylactic removal of the breasts	Out of 1,000 women like you who undergo prophylactic removal of the breasts
Benefits:		
Number of women who would die of breast cancer within 10 years	**20**	**4**
Harms:		
Severe complications during and following the surgery (e.g., infection, impaired healing)	**-**	**100**

Would you consider having prophylactic removal of your breasts?

O yes O no

[Debriefing]

All of the scenarios you were just shown are hypothetical.

Thus, presented numbers about the risk of dying of breast cancer without prophylactic removal of breasts as well as about the potential benefits and harms of the prophylactic removal do not correspond to real numbers derived from clinical studies.

We systematically varied the risk of dying of breast cancer without prophylactic removal of breasts as well as of the potential effects of prophylactic removal as shown in the following table, in order to better understand if and at what benefit-harm-balance women were prepared to consider the prophylactic removal of breasts.

Risk of dying of breast cancer without prophylactic removal of breasts	Risk of dying of breast cancer with prophylactic removal of breasts (weak risk reduction)	Risk of dying of breast cancer with prophylactic removal of breasts (strong risk reduction)
5 / 1000	3 / 1000	1 / 1000
10 / 1000	6 / 1000	2 / 1000
20 / 1000	12 / 1000	4 / 1000

If you need more information, please check, for instance:
www.forecee.eu
www.eveappeal.org.uk
www.cancerresearchuk.org.
www.rki.de

Thank you very much for participating!

Max Planck Institute for Human Development, Harding Center for Risk Literacy

Lentzeallee 94

14195 Berlin(Germany)

Scientific project lead: O. Wegwarth / G. Gigerenzer

Contact: forecee@mpib-berlin.mpg.de
