# Supplementary material for: Do cancer risk and benefit–harm ratios influence women’s consideration of risk-reducing mastectomy? A scenario-based experiment in five European countries
Source: PLoS One. 2019 Jun 12;14(6):e0218188. doi: 10.1371/journal.pone.0218188 (PMC6561593; doi:10.1371/journal.pone.0218188)
Supplement: S6 Fig — (PDF) [file pone.0218188.s009.pdf]

Questionario

<u>Informazioni demografiche:</u>

1) Quanti anni ha?
____

2) Qual è il titolo di studio più elevato da lei conseguito?
- nessuno
- diploma di scuola media inferiore
- diploma di maturità o equivalente
- diploma di laurea

3) Le è mai stato diagnosticato il cancro?
- Sì
- no
- non so

  Se sì: quale tipo di cancro:____________

4) A un membro della sua famiglia ristretta (ad es. genitori, partner, figli) è mai stato diagnosticato un qualsiasi tipo di cancro?
- sì
- no
- non so

  Se sì: quale tipo di cancro:____________

<u>Per comprendere il rischio individuale di cancro e la diagnosi precoce</u>

1) Immagini 1.000 donne della sua età *[Qui includere automaticamente l'età più vicina a quella dell'intervistata]*.
Quante di queste 1.000 donne nei prossimi 10 anni svilupperanno le seguenti forme tumorali?

Cancro al seno:	___ donne su 1.000
Cancro all'ovaio:	___ donne su 1.000
Cancro al collo all'utero:	___ donne su 1.000
Cancro all'utero:	___ donne su 1.000

2) Nel seguente elenco indichi con una crocetta l'affermazione che ritiene corretta (solo una risposta è giusta).

Gli esami di diagnosi precoce come la mammografia...
- apportano vantaggi, come una ridotta mortalità per cancro, senza provocare svantaggi.
- apportano vantaggi, come una ridotta mortalità per cancro, e svantaggi come diagnosi e trattamenti non necessari.
- aiutano a prevenire il cancro, che viene così scoperto prima della sua comparsa.

Percezione generale del rischio

1) In confronto alla possibilità di ricevere una diagnosi di osteoporosi nei prossimi 10 anni, secondo lei quante probabilità esistono che nello stesso periodo di tempo le venga diagnosticato un tumore al seno?

x----------------x--------------x--------------x---------------x
1 2 3 4 5
(Assai meno probabile) (Ugualmente probabile) (Assai più probabile)

2) In confronto alla possibilità di ricevere una diagnosi di cancro al collo dell'utero nei prossimi 10 anni, secondo lei quante probabilità esistono che nello stesso periodo di tempo le venga diagnosticato un tumore al seno?

x----------------x--------------x--------------x---------------x
1 2 3 4 5
(Assai meno probabile) (Ugualmente probabile) (Assai più probabile)

Domande sul test WID

Per la comprensione del test WID:

1) Indichi con una crocetta i tipi di cancro interessati dal test WID (solo una risposta è giusta).

- Cancro all'ovaio, cancro al collo dell'utero, cancro ai polmoni e cancro al pancreas
- Cancro all'intestino, cancro al seno, cancro al collo dell'utero e cancro al fegato
- Cancro al seno, cancro all'ovaio, cancro all'utero e cancro al collo dell'utero

2) Come potrebbe comportarsi una donna, apprendendo dall'esito del test di avere un livello di rischio inferiore alla media (solo una risposta è giusta)?

- Potrebbe ridurre il rischio di falsi allarmi e trattamenti non necessari, sottoponendosi meno frequentemente a esami di diagnosi precoce.
- Potrebbe ridurre il rischio di morte per cancro, sottoponendosi più frequentemente a esami di diagnosi precoce.
- Potrebbe essere certa di non sviluppare mai uno dei tipi di cancro oggetto del test.

3) Come potrebbe comportarsi una donna, apprendendo dall'esito del test di avere un livello di rischio superiore alla media (solo una risposta è giusta)?

- Potrebbe ridurre il rischio di morte per cancro, sottoponendosi meno frequentemente a esami di diagnosi precoce.
- Potrebbe ridurre il rischio di morte per cancro, sottoponendosi più frequentemente a esami di diagnosi precoce o a terapia farmacologica preventiva.
- Potrebbe essere certa di sviluppare uno dei tipi di cancro oggetto del test.

4) Il test WID ha il compito di prevedere il rischio individuale di una donna di sviluppare determinati tipi di tumore specificamente femminili mediante l'analisi dell'epigenoma. Quale delle seguenti affermazioni sull'epigenoma è corretta (solo una risposta è giusta)?

- L'ambiente e il suo stile di vita modificano l'epigenoma delle sue cellule.
- L'epigenoma rimane immutato per tutta la vita.
- L'epigenoma è una cellula con mutazioni cancerogene.

<u>Opinioni e intenzioni riguardo il test WID</u>

Il test WID ha il compito di prevedere il suo rischio individuale di sviluppare uno o più dei quattro tipi di tumore femminile rappresentati da cancro al seno, cancro all'ovaio, cancro al collo dell'utero e cancro all'utero.

1) Riflettendo sul test WID, come ritiene il rapporto tra vantaggi e svantaggi?

x--------------------x------------------------x---------------------x----------------------x

1	2	3	4	5
Gli svantaggi superano chiaramente i vantaggi		Svantaggi e vantaggi sono equilibrati		I vantaggi superano chiaramente gli svantaggi

2) Alcune donne preferirebbero conoscere il loro eventuale rischio di ammalarsi in futuro, mentre altre no. Considerando il test WID, vorrebbe conoscere il suo rischio di sviluppare uno o più dei quattro tipi di tumore femminile nei prossimi 10 anni?

Per il cancro al seno	O sì O no
Per il cancro all'utero	O sì O no
Per il cancro al collo dell'utero	O sì O no
Per il cancro all'ovaio	O sì O no

3) Se il test WID fosse oggi già disponibile e utilizzabile, vi si sottoporrebbe per determinare il suo rischio di sviluppare i quattro tipi di tumore?

- Sicuramente NON mi sottoporrei al test.
- Probabilmente NON mi sottoporrei al test.
- Probabilmente mi sottoporrei al test.
- Sicuramente mi sottoporrei al test.

4) Negli ultimi mesi abbiamo chiesto a diversi gruppi di donne che cosa pensano del test WID.

Il seguente elenco contiene le motivazioni più importanti delle donne intervistate, che si sono dichiarate ***A FAVORE*** di una partecipazione al test. Assegni un numero a tutti i motivi, che anche per lei personalmente sarebbero ***a favore*** del test, ordinandoli in base al significato che rivestono per lei e indicando con "1" il motivo più importante. Qualora due o più motivi abbiano per lei lo

stesso significato, può utilizzare più volte lo stesso numero. Se nessuno dei motivi elencati le appare importante, non assegni alcun numero.

Sottopormi al test...

- diminuirebbe la mia preoccupazione di ammalarmi di cancro.
- mi porterebbe a vivere in maniera più consapevole, ad esempio adottando uno stile di vita più sano.
- aumenterebbe la sensazione di controllo sulla mia vita (ad es. confrontarmi già OGGI con il rischio, prima dell'insorgere del cancro).
- mi sarebbe di aiuto nella scelta della mia strategia in ambito di assistenza medica (ad es. frequenza personalizzata degli esami di diagnosi precoce), per prevenire in maniera ottimale la comparsa del cancro o la morte per cancro.
- mi sarebbe d'aiuto nello sviluppo precoce di strategie volte alla gestione di un rischio superiore alla media.

Il seguente elenco contiene le motivazioni più importanti delle donne intervistate, che si sono dichiarate ***CONTRO*** una partecipazione al test. Assegni un numero a tutti i motivi, che anche per lei personalmente sarebbero ***contro*** il test, ordinandoli in base al significato che rivestono per lei e indicando con "1" il motivo più importante. Qualora due o più motivi abbiano per lei lo stesso significato, può utilizzare più volte lo stesso numero. Se nessuno dei motivi elencati le appare importante, non assegni alcun numero.

Sottopormi al test...

- mi sembrerebbe insensato, perché il suo esito è solo un valore stimato, non in grado di dirmi se svilupperò o meno il cancro.
- in caso di rischio superiore alla media mi darebbe la sensazione di essere colpevole o responsabile dell'esito, per la presenza di una correlazione tra il risultato e il mio stile di vita in passato.
- in caso di un rischio superiore alla media causerebbe preoccupazioni inutili alla mia famiglia e a me e influenzerebbe negativamente la mia attuale qualità della vita.
- in caso di un rischio superiore alla media mi sentirei costretta ad adottare uno stile di vita più salutare o anche a sottopormi a esami di diagnosi precoce con maggiore frequenza.
- in caso di un rischio superiore alla media vivrei con il fiato sospeso in attesa di sviluppare il cancro.

5) *[Sullo schermo compaiono automaticamente i motivi con segno di spunta]* Qui può vedere i motivi da lei selezionati ***A FAVORE*** dell'esecuzione del test WID. Osservandoli, ne identifica UNO talmente forte da superare tutte gli altri?

   _ Sì

      - Se sì, il motivo decisivo è: _________

   _ No, in ogni caso terrei in considerazione tutte i motivi da me selezionati nell'elenco.

6) *[Sullo schermo compaiono automaticamente i motivi con segno di spunta]* Qui può vedere i motivi da lei selezionati ***CONTRO*** l'esecuzione del test WID. Osservandoli, ne identifica UNO talmente forte da superare tutte gli altri?

_ Sì

- Se sì, il motivo decisivo è: __________

_ No, in ogni caso terrei in considerazione tutte i motivi da me selezionati nell'elenco.

<u>Valutazione del rapporto vantaggi/svantaggi:</u>

Le donne con un rischio elevato di sviluppare il cancro al seno possono ricorrere all'asportazione preventiva delle mammelle (mastectomia), per ridurre le probabilità di ammalarsi e morire. Naturalmente questo intervento chirurgico comporta però al tempo stesso anche potenziali svantaggi.

Immagini di avere un rischio di cancro superiore alla media e di avere la possibilità di ricorrere alla mastectomia. Che portata dovrebbero avere per lei i vantaggi, per compensare gli svantaggi potenziali? Consideri i seguenti scenari ***ipotetici***, indicanti differenti rapporti vantaggi/svantaggi. Per ogni scenario indichi se prenderebbe in considerazione o meno l'asportazione preventiva delle mammelle.

	Su 1.000 donne come lei, che non ricorrono all’asportazione preventiva delle mammelle	Su 1.000 donne come lei, che ricorrono all’asportazione preventiva delle mammelle
Vantaggi:		
Numero delle donne che nei prossimi 10 anni morirebbero di cancro al seno	**10**	**2**
Svantaggi:		
Gravi complicazioni durante e dopo l’intervento (ad es. infezione, cattiva guarigione delle ferite)	**-**	**100**

Prenderebbe in considerazione la possibilità di sottoporsi ad asportazione preventiva delle mammelle?

O sì O no

	Su 1.000 donne come lei, che non ricorrono all'asportazione preventiva delle mammelle	Su 1.000 donne come lei, che ricorrono all'asportazione preventiva delle mammelle
Vantaggi:		
Numero delle donne che nei prossimi 10 anni morirebbero di cancro al seno	**10**	**6**
Svantaggi:		
Gravi complicazioni durante e dopo l'intervento (ad es. infezione, cattiva guarigione delle ferite)	**-**	**100**

Prenderebbe in considerazione la possibilità di sottoporsi ad asportazione preventiva delle mammelle?

O sì O no

	Su 1.000 donne come lei, che non ricorrono all'asportazione preventiva delle mammelle	Su 1.000 donne come lei, che ricorrono all'asportazione preventiva delle mammelle
Vantaggi:		
Numero delle donne che nei prossimi 10 anni morirebbero di cancro al seno	**5**	**3**
Svantaggi:		
Gravi complicazioni durante e dopo l'intervento (ad es. infezione, cattiva guarigione delle ferite)	**-**	**100**

Prenderebbe in considerazione la possibilità di sottoporsi ad asportazione preventiva delle mammelle?

O sì O no

	Su 1.000 donne come lei, che non ricorrono all'asportazione preventiva delle mammelle	Su 1.000 donne come lei, che ricorrono all'asportazione preventiva delle mammelle
Vantaggi:		
Numero delle donne che nei prossimi 10 anni morirebbero di cancro al seno	**5**	**1**
Svantaggi:		
Gravi complicazioni durante e dopo l'intervento (ad es. infezione, cattiva guarigione delle ferite)	**-**	**100**

Prenderebbe in considerazione la possibilità di sottoporsi ad asportazione preventiva delle mammelle?

O sì O no

	Su 1.000 donne come lei, che non ricorrono all'asportazione preventiva delle mammelle	Su 1.000 donne come lei, che ricorrono all'asportazione preventiva delle mammelle
Vantaggi:		
Numero delle donne che nei prossimi 10 anni morirebbero di cancro al seno	**20**	**12**
Svantaggi:		
Gravi complicazioni durante e dopo l'intervento (ad es. infezione, cattiva guarigione delle ferite)	**-**	**100**

Prenderebbe in considerazione la possibilità di sottoporsi ad asportazione preventiva delle mammelle?

O sì O no

	Su 1.000 donne come lei, che non ricorrono all'asportazione preventiva delle mammelle	Su 1.000 donne come lei, che ricorrono all'asportazione preventiva delle mammelle
Vantaggi:		
Numero delle donne che nei prossimi 10 anni morirebbero di cancro al seno	**20**	**4**
Svantaggi:		
Gravi complicazioni durante e dopo l'intervento (ad es. infezione, cattiva guarigione delle ferite)	**-**	**100**

Prenderebbe in considerazione la possibilità di sottoporsi ad asportazione preventiva delle mammelle?

O sì O no

[Debriefing]

Tutti gli scenari da lei appena elaborati, relativi a vantaggi e svantaggi di una mastectomia, erano puramente ipotetici.

I dati mostrati negli scenari e riguardanti sia il rischio di morte per cancro senza mastectomia sia i vantaggi e gli svantaggi della mastectomia non corrispondono ai dati reali ricavabili da studi clinici attendibili.

Negli scenari abbiamo modificato sistematicamente i numeri relativi sia al rischio di morte per cancro senza mastectomia sia ai vantaggi e agli svantaggi della mastectomia. Il nostro obiettivo era quello di capire meglio quale rapporto debba intercorrere tra vantaggi e svantaggi di una mastectomia per convincere le donne a ricorrere a tale intervento chirurgico.

Ulteriori informazioni sono disponibili ai seguenti indirizzi:
www.forecee.eu
www.eveappeal.org.uk
www.cancerresearchuk.org.
www.rki.de

La ringraziamo per aver partecipato al nostro studio.

Max-Planck-Institut für Bildungsforschung, Harding-Zentrum für Risikokompetenz
(Istituto per la ricerca in ambito di istruzione, Centro Harding per la competenza negli ambiti a rischio)

Lentzeallee 94

14195 Berlino

Responsabili dei progetti scientifici: O. Wegwarth / G. Gigerenzer

Contatto: forecee@mpib-berlin.mpg.de
