# Supplementary material for: Do cancer risk and benefit–harm ratios influence women’s consideration of risk-reducing mastectomy? A scenario-based experiment in five European countries
Source: PLoS One. 2019 Jun 12;14(6):e0218188. doi: 10.1371/journal.pone.0218188 (PMC6561593; doi:10.1371/journal.pone.0218188)
Supplement: S7 Fig — (PDF) [file pone.0218188.s010.pdf]

Frågeformulär

Demografi:

1) Hur gammal är du?
____

2) Vilken är din högsta uppnådda utbildningsnivå?
- ej avslutad skolgång
- avslutad skolgång utan studentexamen
- Studentexamen eller likvärdigt
- Högskoleexamen

3) Har du någon gång diagnostiserats med cancer?
- ja
- nej
- vet ej
  Om ja: vilken typ av cancer:___________

4) Har någon medlem av din nära familj (t.ex. föräldrar, partner, barn) diagnostiserats med någon typ av cancer ?
- ja
- nej
- vet ej
  Om ja: vilken typ av cancer:___________

Förståelse av personlig cancerrisk och screening

1) Föreställ dig 1000 kvinnor i din ålder*[Include age that is closest ton (to) the age av respondent automatically here]*.
Hur många av dessa 1000 kvinnor kommer inom de kommande 10 åren utveckla följande cancersjukdomar?

Bröstcancer:	___ av 1000 kvinnor
Äggstockscancer:	___ av 1000 kvinnor
Livmoderhalscancer:	___ av 1000 kvinnor
Livmodercancer:	___ av 1000 kvinnor

2) V.g. kryssa för det påstående i följande lista som du anser är sant (endast ett svar är rätt).

Att delta i cancerscreening med metoder såsom mammografi...
- medför nytta såsom minskad cancerdödlighet men inga skador.
- medför nytta såsom en minskad cancerdödlighet och skador såsom onödiga diagnoser och onödiga behandlingar.
- hjälper till att förebygga cancer, eftersom det upptäcker cancer innan detta uppstår

<u>Allmän riskmedvetenhet</u>

1) I jämförelse med sannolikheten att få en benskörhetsdiagnos inom de kommande 10 åren, hur sannolikt är det enligt din uppfattning att inom de kommande 10 åren få en bröstcancerdiagnos?

x----------------x--------------x--------------x---------------x
1 2 3 4 5
(Mycket mindre sannolikt) (Lika sannolikt) (Mycket mer sannolikt)

2) I jämförelse med sannolikheten att få en livmoderhalscancerdiagnos inom de kommande 10 åren, hur sannolikt är det enligt din uppfattning att inom de kommande 10 åren få en bröstcancerdiagnos?

x----------------x--------------x--------------x---------------x
1 2 3 4 5
(Mycket mindre sannolikt) (Lika sannolikt) (Mycket mer sannolikt)

<u>Frågor om WID-testet</u>

<u>Förståelse för WID-testet:</u>

1) Kryssa för den grupp av cancertyper som WID-testet avser (endast ett svar är rätt).

- Äggstockscancer, livmodercancer, lungcancer och bukspottkörtelscancer
- Tjock- och ändtarmscancer, bröstcancer, livmoderhalscancer och levercancer
- Bröstcancer, äggstockscancer, livmodercancer och livmoderhalscancer

2) Vad skulle en kvinna som har ett testresultat som säger hon har en risk under genomsnittet kunna göra (endast ett svar är rätt)?

- Hon skulle kunna minska sin risk att drabbas av falsklarm och onödiga behandlingar genom att delta mer sällan i screening.

- Hon skulle kunna minska sin risk att dö i cancer genom att delta mer ofta i screening.

- Hon kunde känna sig säker på att hon definitivt inte kommer att få någon av de testade cancertyperna.

3) Vad skulle en kvinna som har ett testresultat som säger hon har en risk över genomsnittet kunna göra (endast ett svar är rätt)?

- Hon skulle kunna minska sin risk att dö i cancer genom att delta mer sällan i screening.

- Hon skulle kunna minska sin risk att dö i cancer genom att delta mer ofta i screening, eller ta förebyggande medicinering.

- Hon kunde känna sig säker på att hon definitivt kommer att få någon av de testade cancertyperna.

4) WID-testet ska förutsäga en kvinnas personliga risk att utveckla olika kvinnocancertyper genom att analysera hennes epigenom. Vilket av följande påståenden om epigenomet är korrekt (endast ett svar är rätt)?

- Din omgivning och din livsstil förändrar epigenomet i dina celler.
- Epigenomet förblir alltid oförändrat under hela livet.
- Epigenomet är en cell med cancermutationer.

<u>Attityder och intentioner beträffande WID-testet</u>

WID-testet är avsett att förutsäga din personliga risk för en eller flera av de fyra kvinnocancertyperna: Bröstcancer, äggstockscancer, livmoderhalscancer, livmodercancer

1) När du tänker på WID-testet, hur känner du då inför förhållandet mellan nytta och skador?

x-------------------x-----------------------x--------------------x---------------------x

1	2	3	4	5
Skadorna överväger klart nyttan		Skadorna och nyttan är lika		Nyttan överväger klart skadorna

2) Medan vissa kvinnor vill känna till sin risk att insjukna i framtiden, vill andra kvinnor inte veta detta. När du tänker på WID-testet, skulle du vilja veta din risk att insjukna i en eller flera av de fyra kvinnocancertyperna inom de kommande 10 åren?

För bröstcancer	O ja O nej
För livmodercancer	O ja O nej
För livmoderhalscancer	O ja O nej
För äggstockscancer	O ja O nej

3) Om WID-testet idag var enkelt genomförbart och fritt tillgängligt, skulle du låta göra ett sådant, för att ta reda på din risk att drabbas av någon av de fyra cancertyperna?

   - Jag skulle definitivt INTE göra testet.
   - Jag skulle sannolikt INTE göra testet.
   - Jag skulle sannolikt göra testet
   - Jag skulle definitivt göra testet.

4) De senaste månaderna har vi frågat olika grupper med kvinnor om deras tankar beträffande WID-testet.

Följande lista visar de viktigaste skälen för de tillfrågade kvinnorna som talade ***FÖR*** ett deltagande i testet. Ange för alla skäl som för dig personligen också talar ***för*** testet, en siffra i relation till betydelsen för dig. Börja här med en 1:a för det viktigaste skälet. I det fallet att två eller flera skäl är lika viktiga för dig, ge dem samma siffra flera gånger. Om något av de listade skälen inte har någon betydelse för dig, ge det då ingen siffra.

Att göra testet...

- skulle minska min oro att få cancer.
- skulle leda till att jag levde mer medvetet, genom att vidta åtgärder som att exempelvis skaffa mig en sundare livsstil.
- skulle öka kontrollen jag upplever att jag har över mitt liv (t.ex. genom att diskutera risken att potentiellt diagnosticeras med sjukdomen redan innan den uppstår).
- skulle stötta mig i valet av min medicinska strategi (t.ex. personligt anpassad frekvens av screening), för att på bästa möjliga vis förebygga att insjukna eller dö i cancer.
- skulle hjälpa mig att i ett tidigt skede utveckla strategier för att kunna hantera en risk som är högre än genomsnittet.

Följande lista visar de viktigaste skälen för de tillfrågade kvinnorna som talade ***MOT*** ett deltagande i testet. Ange för alla skäl som för dig personligen också talar ***mot*** testet, en siffra i relation till betydelsen för dig. Börja här med en 1:a för det viktigaste skälet. I det fall att två eller flera skäl är lika viktiga för dig, ge dem samma siffra flera gånger. Om något av de listade skälen inte har någon betydelse för dig, ge det då ingen siffra.

Att göra testet...

- skulle jag betrakta som meningslöst, eftersom testresultatet endast är en uppskattning som inte säger mig något om huruvida jag verkligen kommer att få cancer.
- skulle, ifall jag fick en risk över genomsnittet, kunna ge mig känslan av att vara skyldig eller ansvarig för resultatet, eftersom detta ger en koppling mellan testresultatet och min tidigare livsstil.
- Skulle, ifall jag fick en risk över genomsnittet, i onödan oroa mig och min familj och negativt påverka min livskvalitet.
- Skulle, ifall jag fick en risk över genomsnittet, sätta press på mig att anpassa min livsstil till min risk eller att behöva låta göra fler screeningundersökningar.
- Skulle, ifall jag fick en risk över genomsnittet, få mig att ständigt förvänta mig en cancer.

5) *[Screen presents ticked reasons automatically]* Här ser du skälen, som du angett ***FÖR*** att genomföra WID-testet. När du tittar på detta, finns det ETT skäl som väger så tungt att det överskuggar alla andra skäl att genomföra testet?

_ ja

- Om ja, vilket är beslutsskälet: __________

_ Nej, jag skulle definitivt beakta alla skäl som jag angett på min lista.

6) *Screen presents ticked reasons automatically]* Här ser du skälen, som du angett ***MOT*** att genomföra WID-testet. När du tittar på detta, finns det ETT skäl som väger så tungt att det överskuggar alla andra skäl?

_ ja

- Om ja, vilket är beslutsskälet: __________

_ Nej, jag skulle definitivt beakta alla skäl som jag angett på min lista.

Värdering av förhållandet mellan nytta och skador:

Kvinnor med högre bröstcancerrisk kan låta operera bort brösten i förebyggande syfte (mastektomi), för att minska risken för att insjukna och eventuellt dö i bröstcancer. Trots att risken att insjukna eller dö i bröstcancer kan minskas genom mastektomi, medför detta kirurgiska ingrepp även potentiella skador.

Föreställ dig att du har en cancerrisk över genomsnittet och du erbjuds möjligheten till en mastektomi. Hur stor måste nyttan vara för dig för att uppväga de potentiella skadorna? Gå igenom följande ***hypotetiska*** scenarier som visar olika förhållanden mellan nytta och skador. Ange för varje scenario om du för att minska din risk att dö i bröstcancer skulle överväga att i förebyggande syfte operera bort brösten eller inte.

	Av 1000 kvinnor som du som inte i förebyggande syfte låter operera bort brösten	Av 1000 kvinnor som du som i förebyggande syfte låter operera bort brösten
Nytta:		
Antal kvinnor som inom 10 år skulle dö i bröstcancer	**10**	**2**
Skador:		
Svåra komplikationer under och efter operationen (t.ex. infektioner, dålig sårläkning)	**-**	**100**

Skulle du överväga att i förebyggande syfte låta operera bort brösten?

O ja O nej

	Av 1000 kvinnor som du som inte i förebyggande syfte låter operera bort brösten	Av 1000 kvinnor som du som i förebyggande syfte låter operera bort brösten
Nytta:		
Antal kvinnor som inom 10 år skulle dö i bröstcancer	**10**	**6**
Skador:		
Svåra komplikationer under och efter operationen (t.ex. infektioner, dålig sårläkning)	**-**	**100**

Skulle du överväga att i förebyggande syfte låta operera bort brösten?

O ja O nej

	Av 1000 kvinnor som du som inte i förebyggande syfte låter operera bort brösten	Av 1000 kvinnor som du som i förebyggande syfte låter operera bort brösten
Nytta:		
Antal kvinnor som inom 10 år skulle dö i bröstcancer	**5**	**3**
Skador:		
Svåra komplikationer under och efter operationen (t.ex. infektioner, dålig sårläkning)	**-**	**100**

Skulle du överväga att i förebyggande syfte låta operera bort brösten?

O ja O nej

	Av 1000 kvinnor som du som inte i förebyggande syfte låter operera bort brösten	Av 1000 kvinnor som du som i förebyggande syfte låter operera bort brösten
Nytta:		
Antal kvinnor som inom 10 år skulle dö i bröstcancer	**5**	**1**
Skador:		
Svåra komplikationer under och efter operationen (t.ex. infektioner, dålig sårläkning)	**-**	**100**

Skulle du överväga att i förebyggande syfte låta operera bort brösten?

O ja O nej

	Av 1000 kvinnor som du som <u>inte</u> i förebyggande syfte låter operera bort brösten	Av 1000 kvinnor som du som i förebyggande syfte låter operera bort brösten
Nytta:		
Antal kvinnor som inom 10 år skulle dö i bröstcancer	**20**	**12**
Skador:		
Svåra komplikationer under och efter operationen (t.ex. infektioner, dålig sårläkning)	**-**	**100**

Skulle du överväga att i förebyggande syfte låta operera bort brösten?

O ja O nej

	Av 1000 kvinnor som du som inte i förebyggande syfte låter operera bort brösten	Av 1000 kvinnor som du som i förebyggande syfte låter operera bort brösten
Nytta:		
Antal kvinnor som inom 10 år skulle dö i bröstcancer	**20**	**4**
Skador:		
Svåra komplikationer under och efter operationen (t.ex. infektioner, dålig sårläkning)	**-**	**100**

Skulle du överväga att i förebyggande syfte låta operera bort brösten?

O ja O nej

[Debriefing]

Alla de scenarier om nytta och skador med en mastektomi som presenterats har varit hypotetiska.

De i scenarierna angivna siffrorna om risken för cancerdödsfall utan mastektomi, samt om nyttan och skadorna med mastektomi, bygger inte på verkliga siffror från kliniska studier.

Vi förändrade i scenarierna systematiskt siffrorna om cancerdödsfallsrisken utan mastektomi samt nyttan och skadorna med mastektomi. Vårt syfte var här att bättre förstå i vilket förhållande nyttan och skadorna med mastektomi behöver stå, för att kvinnor skulle överväga ingreppet.

Om du behöver mer information, hittar du denna t.ex. under:
www.forecee.eu
www.eveappeal.org.uk
www.cancerresearchuk.org.
www.rki.de

Vi tackar för ditt deltagande i vår studie.

Max-Planck-Institutet für utbildningsforskning, Harding-centret för riskkompetens

Lentzeallee 94

14195 Berlin

Vetenskaplig projektledning: O. Wegwarth / G. Gigerenzer

Kontakt: forecee@mpib-berlin.mpg.de
