# Supplementary material for: Do cancer risk and benefit–harm ratios influence women’s consideration of risk-reducing mastectomy? A scenario-based experiment in five European countries
Source: PLoS One. 2019 Jun 12;14(6):e0218188. doi: 10.1371/journal.pone.0218188 (PMC6561593; doi:10.1371/journal.pone.0218188)
Supplement: S1 Text — (PDF) [file pone.0218188.s011.pdf]

***S1 Text.* Sampling frame and survey administration**

The Harris Interactive Panel and the Toluna Panel are representative of the general population with respect to age, education, gender and regions in each of the countries and comprise about 78,000 to 275,000 female participants of the age group targeted in this study, respectively. Participants have agreed in advance to be invited to online studies.

In January 2017, Harris Interactive contacted eligible panellists (based on gender, age and education characteristics) in each of the five European countries via email. The email provided basic information about the study, the link to the survey including information leaflet (with a personalised password) and an offer of a five-euro honorarium upon completion of the survey. Each survey and leaflet was presented in the respective language of the target population in the five countries. Up to two reminder e-mails were sent to non-respondents until the intended sample size was met: 311 participants per country, for an expected R square of .07 of up to eight predictors in a multiple regression model.
